# Biosynthetic CircRNA_001160 induced by PTBP1 regulates the permeability of BTB via the CircRNA_001160/miR-195-5p/ETV1 axis

**DOI:** 10.1038/s41419-019-2191-z

**Published:** 2019-12-20

**Authors:** Hua Li, Shuyuan Shen, Xuelei Ruan, Xiaobai Liu, Jian Zheng, Yunhui Liu, Chunqing Yang, Di Wang, Libo Liu, Jun Ma, Teng Ma, Ping Wang, Heng Cai, Zhen Li, Lini Zhao, Yixue Xue

**Affiliations:** 1 Department of Neurobiology, College of Basic Medicine, China Medical University, Shenyang, 110122 People’s Republic of China; 2 Key Laboratory of Cell Biology, Ministry of Public Health of China, China Medical University, Shenyang, 110122 People’s Republic of China; 3 Key Laboratory of Medical Cell Biology, Ministry of Education of China, China Medical University, Shenyang, 110122 People’s Republic of China; 4 Department of Neurosurgery, Shengjing Hospital of China Medical University, Shenyang, 110004 People’s Republic of China; 5 Liaoning Clinical Medical Research Center in Nervous System Disease, Shenyang, 110004 People’s Republic of China; 6 Key Laboratory of Neuro-oncology in Liaoning Province, Shenyang, 110004 People’s Republic of China; 7 Department of pharmacology, Shenyang Medical College, Shenyang, 110034 People’s Republic of China

**Keywords:** Mechanisms of disease, miRNAs

## Abstract

The presence of the blood-tumor barrier (BTB) severely impedes the transport of anti-neoplasm drugs to the central nervous system, affecting the therapeutic effects of glioma. Glioma endothelial cells (GECs) are the main structural basis of the BTB. Circular RNA is considered to be an important regulator of endothelial cell growth. In this study, we found that polypyrimidine tract binding protein 1 (PTBP1) and circRNA_001160 were remarkably upregulated in GECs. Knockdown of PTBP1 or circRNA_001160 significantly increased BTB permeability, respectively. As a molecular sponge of miR-195-5p, circRNA_001160 attenuated its negative regulation of the target gene ETV1 by adsorbing miR-195-5p. In addition, ETV1 was overexpression in GECs. ETV1 bounded to the promoter regions of tight junction-related proteins and increased the promoter activities, which significantly promoted the expression levels of tight junction-related proteins. The present study showed that the combined application of PTBP1, circRNA_001160, and miR-195-5p with the anti-tumor drug Dox effectively promoted Dox through BTB and extremely induced the apoptosis of glioma cells. Our results demonstrated that the PTBP1/circRNA_001160/miR-195-5p/ETV1 axis was critical in the regulation of BTB permeability and provided new targets for the treatment of glioma.

## Introduction

Glioblastoma (GBM) is the most common primary malignant brain tumor in the adult central nervous system with its highly invasiveness and diffusivity^1^. Despite the use of most conventional therapies such as surgical resection and chemoradiotherapy, patients with glioma still gain poor postoperative outcomes, high recurrence rates, and low survival rates^2^. The blood-tumor barrier (BBB) is composed of normal capillary endothelial cells (ECs), preventing the entry of harmful substances, and is an indispensable vascular barrier to protect the brain^3,4^. Glioma leads to the BBB structure remodeling, forming the blood-tumor barrier (BTB)^5^. The BTB constitutes major obstacles to the transport of therapeutics in brain tumors^6^. Selective opening of the BTB to increase anti-neoplasm drugs concentration in tumor tissues is crucial for the treatment of malignant glioma.

In addition to the lipid-soluble small molecules with a molecular mass under a 400–600-Da threshold, BTB and BBB limit most compounds, making potentially powerful anti-neoplasm drugs unusable for GBM^7^. Microbubble-enhanced diagnostic ultrasound effectively increases the permeability of BTB in glioma by opening KCa channels^8^. In preclinical studies, it was confirmed that inhibition of BMX kinase by ibrutinib in vivo strongly destroys tumor pericytes, and selectively damages BTB instead of BBB, thereby increasing drug effusion into established tumors and enhancing the chemotherapeutic efficacy of drugs with poor BTB penetration^3^. In addition, in targeted therapy of molecules, researches show that, low doses of EMAP-II induces high permeability of BTB through the cAMP/PKA/Rac1 signaling pathway^9^. Single or combination therapy with KHDRBS3, cDENND4C, and miR-577 is effective in inducing apoptosis of glioma cells by promoting the anti-neoplasm drug doxorubicin across BTB^10^.

RNA-binding proteins (RBPs) are involved in multiple steps of post-transcriptional regulation of cellular biological process, including pre-mRNA splicing, RNA editing, and polyadenylation^11^. Polypyrimidine tract binding protein 1 (PTBP1) is located at 19p13.3 and is one of the most widely studied splicing regulatory factors in the hnRNP family^12^. PTBP1 is a key protein that regulates precursor mRNA splicing and alternative splicing events^13^. PTBP1 promotes the expression conversion of two pyruvate kinases PKM1 and PKM2, and enhances the aerobic glycolysis of cells, thereby promoting the proliferation of glioma cells^14^. In addition, PTBP1 shows high expression level and participates in the malignant biological behaviors of bladder cancer^15^, colon cancer^16^, and breast cancer^17^ cells. The study of PTBP1 on the regulation of BTB permeability has not been reported yet.

Circular RNAs (circRNAs) consist of a covalently closed loop without a 5'-terminal cap and 3'-terminal poly A tail, which are more stable than long non-coding RNAs^18^. CircRNAs have a variety of biological functions. For example, circRNAs act as molecular sponges for microRNAs;^19^ post-transcriptional regulation of gene expression;^20^ and encode polypeptides involved in protein translation^21^. CircRNA_001160 (hsa_circ_0001417, circ-ANKRD17) is located on chromosome 14 and has a full length of 1832 bp. CircRNA_001160 is derived from the cyclization of linear RNA ANKRD17 (ankyrin repeat domain 17). ANKRD17 plays a vital role in liver and cardiovascular development as well as cell cycle regulation^22^. In addition, ANKRD17 is critical for vascular integrity during embryonic development^23^. At present, there is no report on the expression and function of circRNA_001160 in GECs.

MicroRNAs (miRNAs) are highly conserved single stranded non-coding RNAs^24^. MiRNAs regulate target gene expression by inducing mRNA degradation or blocking protein translation^25^. The regulation of miRNAs mediated BBB damage therapy can improve vascular cognitive impairment^26^. Numerous studies demonstrated that miRNAs could regulate BTB permeability^27,28^. Overexpression of miR-195-5p could reduce angiogenesis of ovarian cancer^29^. In addition, miR-195-5p is downregulated in glioma tissues, and upregulated miR-195-5p suppresses the malignant biological behaviors of glioma cells^30^. Bioinformatics software starBase v2.0 was used to predict that miR-195-5p binding sites existed on circRNA_001160. However, the expression of miR-195-5p in GECs and the underlying molecular mechanism of circRNA_001160/miR-195-5p are unclear.

Ets variant gene 1 (ETV1), also known as ER81, is a member of the E-26 (ETS) transcription factor family^31^. ETS factors are transcription factors that bind to promoters and enhancers to recruit other transcriptional components^32^. ETV1 has been shown to regulate the biological processes of tumor cells as an oncogene in Ewing's sarcoma^33^, melanoma^34^, and triple-negative breast cancer^35^.

This study established an in vitro BTB model. Firstly, the endogenous expressions of PTBP1, circRNA_001160, miR-195-5p and ETV1 in astrocyte endothelial cells (AECs) and glioma endothelial cells (GECs) were detected. Furthermore, the above-mentioned molecular interaction mode and the mechanisms of regulating BTB permeability were clarified. This study aimed to demonstrate the role of the PTBP1/circRNA_001160/miR-195-5p/ETV1 pathway in regulating BTB permeability, which can provide a new strategy for the comprehensive treatment for glioma.

## Materials and methods

### Cell lines and cell culture

The immortalized human brain EC line hCMEC/D3 was kindly provided by Dr.Couraud from the Institut Cochin, Paris, France. ECs were developed on culture inserts (0.4-mm pore size; Corning, Lowell, MA, USA) coated with 150 μg/mL Cultrex Rat Collagen I (R&D Systems, Minneapolis, MN, USA). ECs were cultured in endothelial basal medium (EBM-2) (Lonza, Walkersville, MD, USA), containing 5% fetal bovine serum (FBS) “Gold” (PAA Laboratories, Pasching, Austria), 1% penicillin-streptomycin (Life Technologies, Paisley, UK), 1.4 μmol/L hydrocortisone (Sigma-Aldrich, St Louis, MO, USA), 1% chemically defined lipid concentrate (Life Technologies, Paisley, UK), 5 μg/mL ascorbic acid (Sigma-Aldrich), 10 mmol/L HEPES (PAA Laboratories), and 1 ng/mL human basic fibroblast growth factor (bFGF) (Sigma-Aldrich). ECs algebra were maintained at 30–40 generations. Human glioma cell line U87 and human embryonic kidney cell line 293T (HEK293T) were purchased from Shanghai Institutes for Biological Sciences Cell Resource Center and developed in Dulbecco’s modified Eagle’s medium (DMEM) of high glucose with 10% FBS (GIBCO, Carlsbad, CA, USA). Normal human astrocytes (NHAs) were purchased from Sciencell Research Laboratories (Carlsbad, CA, USA) and developed in RPMI-1640 culture medium (GIBCO, Grand Island, NY, USA) with 10% FBS. All cells were maintained at 37 °C, 5% CO_2_, in a humidified incubator.

### Establishment of in vitro BTB and BBB model

In vitro BTB and BBB models were established by co-culture of EC and U87 cells. For the BTB model in vitro, the U87 cells were inoculated in six-well plates at 2 × 10^4^ cells/well, and culture medium was developed for 2 days. After culturing U87 cells for 2 days, ECs were inoculated in the upper compartment at 2 × 10^5^ cells/well on the upper side of inserts pre-coated with Cultrex Rat Collagen I (R&D Systems, Minneapolis, MN, USA). EC and U87 cells were cultured with prepared EBM-2 medium, and the medium was updated every 2 days. A GEC model EC phenotype obtained in vivo gliomas was obtained after 4 days of co-culture. For the BBB model in vitro, ECs were co-cultured with NHA using the same method described above. An AEC model EC phenotype obtained in vivo gliomas was obtained after 4 days of co-culture.

### Quantitative real-time PCR (qRT-PCR) assays

Total RNAs were extracted from AECs and GECs with Trizol reagent (Life Technologies, Carlsbad, CA, USA), according to the manufacturer’s description. RNA concentration and quality were measured by the 260/280 nm ratio using Nanodrop Spectrophotometer (ND-100; Thermo Fisher Scientific, Waltham, MA, USA). One Step SYBR Prime Script RT-PCR Kit (Takara Biomedical Technology, Dalian, China) was used to identify the expression levels of PTBP1 (NM_002819.5), linear ANKRD17 (NM_032217.5), ETV1 (NM_004956.5). One Step PrimeScript™ RT-PCR Kit (Takara) was used to identify the expression level of circRNA_001160 (NM_198889). In addition, RNase R was used to confirm the relative expression of circRNA _001160 and eliminate the effect of linear ANKRD17. GAPDH was used as endogenous control. The design of divergent primer and convergent primer was preformed by Geneseed Biotechnology company (Guangzhou). The expression level of miR-195-5p (NR_029712.1) was detected by TaqMan MicroRNA Reverse Transcription kit and TaqMan Universal Master Mix II (Applied Biosystems). U6 was used as endogenous control. All qRT-PCR reactions were performed using the 7500 Fast RT-PCR System (Applied Biosystems, USA). The relative quantification 2^−ΔΔCt^ method was applied to calculate the expressions of genes. The primers and probes used in this study are shown in Supplementary table S1.

### Cell transfection

Short-hairpin RNAs (shRNAs) directed against human PTBP1, circRNA_001160 and ETV1 gene were constructed in pGPU6/GFP/Neo vector (Gene Pharma, Shanghai, China) to generate silencing plasmids. The human ETV1 gene coding sequence was ligated into the pIRES2-EGFP vector (GeneChem, Shanghai, China) to construct the ETV1 overexpression plasmid. The pGPU6/GFP/Neo and pIRES2-EGFP empty vectors without targeting sequences were used as negative controls (NCs). ECs were cultured into 24-well plates and transfected with plasmids by Opti-MEMI, Lipofectamine LTX and Plus Reagents (Life Technologies) when they reached about 80% confluence. Stable transfected cells were selected using G418 and puromycin (Sigma-Aldrich, St. Louis, MO, USA). G418-resistant (or purinomycin-resistant) cell clones were established at about 4 weeks. For co-transfection of sh-PTBP1 and sh-circRNA_001160, cells with stably knocked down PTBP1 were transfected with sh-circRNA_001160. G418 and purinomycin dual-resistant clone were selected. Furthermore, agomir miR-195-5p (pre-miR-195-5p), antagomir miR-195-5p (anti-miR-195-5p), and their respective NC (Gene Pharma, Shanghai, China) were transfected into ECs using Lipofectamine 3000 Reagents (Life Technologies). Then qRT-PCR and western blot were performed to measure transfection efficiency. For co-transfection of sh-circRNA_001160 and pre/anti-miR-195-5p or overexpressed ETV1 (ETV1) and pre-miR-195-5p, cells that stably sh-circRNA_001160, ETV1, or their respective NC were transiently transfected with pre/anti-195-5p or its NC. The sequences of all sh-RNA templates are shown in Supplementary Table S2. To avoid shRNA-mediated off-target effects, we transfected silencing plasmids at multiple sites and tested transfection efficiency. Then, the shRNA with the best silencing efficiency was selected for functional study. The transfection efficiencies are shown in Additional file 1: Fig. S2.

### Transendothelial electric resistance (TEER) assays

In order to measure the integrity and permeability of BTB, TEER assay was determined by the Millicell-ERS apparatus (Millipore, Billerica, MA, USA) after the successful establishment of an in vitro BTB model. Before each measurement, the TEER assay was recorded at room temperature for 30 min and the culture medium was updated to ensure temperature balance and homogeneity of the culture environment. Through the measurement of resistance barrier property subtracting background, and then multiplied by the effective surface area of membrane to calculate the final resistance (Ω cm^2^).

### Horseradish peroxidase flux (HRP) assays

After the establishment of a normal BBB model for in vitro glioma regulation, 1 mL medium containing 10 mg/mL HRP (0.5 mm, Sigma-Aldrich) was added to the upper system, and 2 mL medium was added to the hole. Then, after incubating for 1 h at 37 °C, 5 μL of medium was collected from the lower compartment. The samples using tetramethyl-benzidine to colorimetric analysis. The absorbance was measured at 370 nm with a spectrophotometer. The final HRP flux was expressed as picomole passed per square centimeter surface area per hour (pmol/cm^2^/h).

### Western blot assays

The total proteins were lysed on ice with RAPI buffer (Beyotime Institute of Biotechnology) and protease inhibitors (10 mg/mL aprotinin, 10 mg/mL phenyl-methylsulfonyl fluoride [PMSF], and 50 mM sodium orthovanadate) for half an hour and then centrifuged at 17,000*g* for 45 min at 4 °C. The amount of the sample was measured using a BCA protein assay kit (Beyotime Institute of Biotechnology, Jiangsu, China). Proteins were transferred to polyvinylidene fluoride (PVDF) membrane by SDS-PAGE electrophoresis. After SDS-PAGE electrophoresis and transfer, the PVDF membrane was blocked with skim milk powder for 2 h at room temperature. Then membranes were incubated with the primary antibodies as follows: PTBP1 (1:1,000; Proteintech, USA), ETV1 (1:1,000, Affinity Biosciences, USA), GAPDH (1:10,000; Proteintech, USA), ZO-1 (1:300; Life Technologies, Frederick, MD, USA), occludin (1:1,000; Proteintech, USA), and claudin-5 (1:300; Life Technologies, Frederick, MD, USA) at 4 °C overnight. The membrane was incubated for 2 h at room temperature with HRP-conjugated secondary antibody. These protein blots were observed with enhanced chemiluminescence kit (ECL) (Santa Cruz Biotechnology, Dallas, TX) and detected by the ECL assay system (Thermo Scientific, Beijing, China). The protein strips were then scanned using Chemi Imager 5500 V2.03 software, and the integrated light density values (IDVs) was calculated by Fluor Chen 2.0 software, with GAPDH as an internal control.

### Immunofluorescence (IF) assays

Cells were fixed with 4% paraformaldehyde for 20 min at room temperature and permeated in PBS containing 0.2% Triton X-100 for 10 min (ZO-1 and claudin-5) or fixed with methanol for 10 min at −20 °C (occludin), followed by incubation in 5% BSA blocking buffer for 2 h at room temperature. Then, cells were incubated with primary antibodies against ZO-1 (1:50; Life Technologies), occludin (1:50; Life Technologies), and claudin-5 (1:50; Life Technologies) overnight at 4 °C. After washing three times with PBS/Tween 20 (PBST), cells were incubated with Alexa-Fluor-488-labeled goat anti-mouse IgG or anti-rabbit IgG secondary antibody (1:500; Beyotime Institute of Biotechnology, Jiangsu, China) for 2 h at room temperature. The nucleus were then counterstained with 0.5 mg/mL DAPI for 5 min. Finally, the staining was observed by confocal microscopy (confocal microscopy parameters: gain value, 2; gamma value, 1; DAPI laser strength, 79%; Alexa Fluor, 68%).

### FISH assays

Used to identify circRNA_001160 and miR-195-5p expression and binding localization in AECs and GECs, circRNA_001160 probe (red-labeled, Biosense, Guangzhou, China) and miR-195-5p probe (green-labeled, Exiqon, Copenhagen, Denmark) were used. In brief, slides were treated with PCR-grade proteinase-K (Roche Diagnostics, Mannheim, Germany) blocked after with prehybridization buffer (3% BSA in 4 × saline-sodium citrate, SSC). The hybridization mix was prepared with circRNA_001160 probe or miR-195-5p probe in hybridization solution. Then the slides were washed with washing buffer. The sections were stained with anti-digoxin rhodamine conjugate (1:100, Exon Biotech Inc, Guangzhou, China) at 37 °C for 1 h away from light. The sections were stained with 4′,6-diamidino-2-phenylindole (DAPI, Beyotime, China) for nuclear staining subsequently. All fluorescence images (100×) were captured using a fluorescence microscope (Leica, Germany).

### Northern blotting

RNAs were isolated from AECs and GECs samples using Trizol reagent (Life Technologies). CircRNA_001160 for RNA imprinting was implemented using a Biotin RNA marker mixture (Roche). RNA samples were isolated by electrophoresis and transferred to NC membranes, which were then incubated with a hydrated buffer containing probes. Finally, the chemiluminescent nucleic acid detection module (Thermo Scientific) was used to detect RNA signals. The measurement was repeated three times.

### Reporter vector construction and dual-luciferase reporter assays

The predicted binding sequences and mutant sequences of miR-195-5p in circRNA_001160 and ETV1-3'-UTR were amplified by qRT-PCR and cloned downstream of the pmir-GLO dual luciferase miRNA target expression vector (Promega, Madison, WI, USA). Construction of a dual luciferase reporter vector (circRNA-miR-Wt/circRNA-miR-Mut1/circRNA-miR-Mut2/circRNA-miR-Mut3 and ETV1-3'-UTR-Wt/ETV1-3'-UTR-Mut, Gene Pharma). HEK293T cells were seeded in 96-well plates. After 24 h, when the cell density was between 60% and 80%, the circRNA-miR-Wt/circRNA-miR-Mut1/circRNA-miR-Mut2/circRNA-miR-Mut3 or ETV1-3'-UT

R-Wt/ETV1-3'-UTR-Mut dual luciferase vector and pre-miR-195-5p (or pre-NC) plasmids using with Lipofectamine 3000 were co-transfected into HEK293T cells. Dual-luciferase activity was measured 48 h after transfection. The dual luciferase reporter assay system (Promega) was used to analyze luciferase activity. Relative luciferase activity was expressed as the ratio of firefly luciferase activity to renilla luciferase activity. In addition, the human full-length ETV1 gene was constructed into pEX3 (pGCMV/MCS/Neo) plasmid vector (GenePharma). HEK293T cells were co-transfected with pGL3 vector (either with wide-type promoter regions or mutated promoter regions) and pEX3-ETV1 (or pEX3 empty vector) using Lipofectamine 3000. The promoter activity of constructed plasmid was normalized with the co-transfected reference vector (pRL-TK) and expressed as relative to the activity of pEX3 empty vector, which the activity set to 1.

### CircRNA, miRNA, and transcription factor microarrays

CircRNA, miRNA, and transcription factor microarray analyses, sample preparation and microarray hybridization were performed by Kangchen Biotechnology Corporation (Shanghai, China).

### Native and nascent RNA immunoprecipitation (RIP) assays

Whole cell lysates from the control group and the anti-miR-195-5p group were incubated with RIP immunoprecipitation buffer containing magnetic beads conjugated with human anti-argonaute2 (Ago2) antibody (Millipore, Billerica, MA, USA) and NC normal mouse IgG (Millipore). The samples were incubated with the protease K buffer and the immunoprecipitated RNA was isolated. In addition, purified RNA was collected and analyzed by qRT-PCR to verify the presence of binding targets.

The wild-type and mutant plasmids were performed by Gene Pharma. The plasmids used with Lipofectamine 3000 were co-transfected into ECs. For nascent RIP, cells were incubated with 100 μM DRB (Sigma, D1916) for 3 h to block Pol II transcription. Transcription was restarted after removal of the DRB and the newly transcribed RNA were labeled with 200 μM 4sU for 30 min (Sigma, T4509) as described above. Native RIP was performed with anti-human PTBP1 (Santa Cruz, USA) and NC normal mouse IgG (Millipore), and the 4su-labeled RNA was purified with RIP products, and the primers for qRT-PCR are performed listed in table S1. The sequences of wild-type and mutant plasmids are listed in Supplementary table S4.

### Chromatin immunoprecipitation (ChIP) assays

The ChIP assays were performed using the Simple Chip Enzymatic Chromatin IP kit (Cell Signaling Technology, Danvers, MA, USA) according to the manufacturer’s instructions. In brief, GECs were cross-linked with EBM-2 containing 1% formaldehyde for 10 min, then glycine was added and incubated at room temperature for 5 min to quench the cross-linking. The cells were collected in a lysis buffer containing PMSF. In addition, the use of micrococcus nuclease digested chromatin at temperature of 37 °C for 20 min and frequently mixed. Immunoprecipitation was incubated with 2 μg anti-ETV1 antibody (Abcam, USA). ProteinG agarose beads were used for immunoprecipitation in each sample and incubated overnight, and they were gently shaken at 4 °C while 2% lysates were used as input reference. Deliver DNA ChIP connected with 5 mol/L NaCl and proteinase K reversed 2 h under 65 °C, and then purified DNA. In each PCR reaction, the corresponding inputs were obtained in parallel for PCR validation. PCR products were isolated from 3% agarose gel. Primers for ChIP PCR are shown in Supplementary table S3.

### Apoptosis analysis

In vitro BTB models were established using transfected sh-PTBP1 cells, sh-circRNA_001160 cells, pre-miR-195-5p cells, sh-PTBP1 + sh-circRNA_001160 cells, sh-circRNA_001160 + pre-miR-195-5p cells. After establishing the in vitro BTB model, 10 μM of Doxorubicin (Beyotime Institute of Biotechnology, Jiangsu, China) was added to the transwell chamber. The normal control group (control) did not join the Dox processing. After 12 h in each group, the U87 cells in the lower chamber were digested to prepare a cell suspension, and the supernatant was discarded after centrifugation. Cell apoptosis was detected by staining with Annexin V-PE/7AAD (Southern Biotech, Birmingham, AL, USA) according to the manufacturer's instructions. Add 500 μl of Binding Buffer suspension cells per tube, add 5 μl PI and 5 μl of FITC were mixed, and reacted at room temperature for 15 min protecting from light. Flow cytometry (FACScan, BD Biosciences) was used to detect apoptosis.

### Statistical analysis

All statistical analyses were carried out using the GraphPad Prism v5.01 (GraphPad, La Jolla, CA). All data were expressed as the mean ± SD. Significant statistical differences between the two groups were determined by Student's *t* test (two-tailed). For three or more groups, one-way ANOVA was used for statistical analysis, followed by post-Dunnett testing. If *P* < 0.05, the difference was considered statistically significant.

## Results

### PTBP1 was upregulated in GECs, and knockdown of PTBP1 increased BTB permeability

**Fig. 1:**
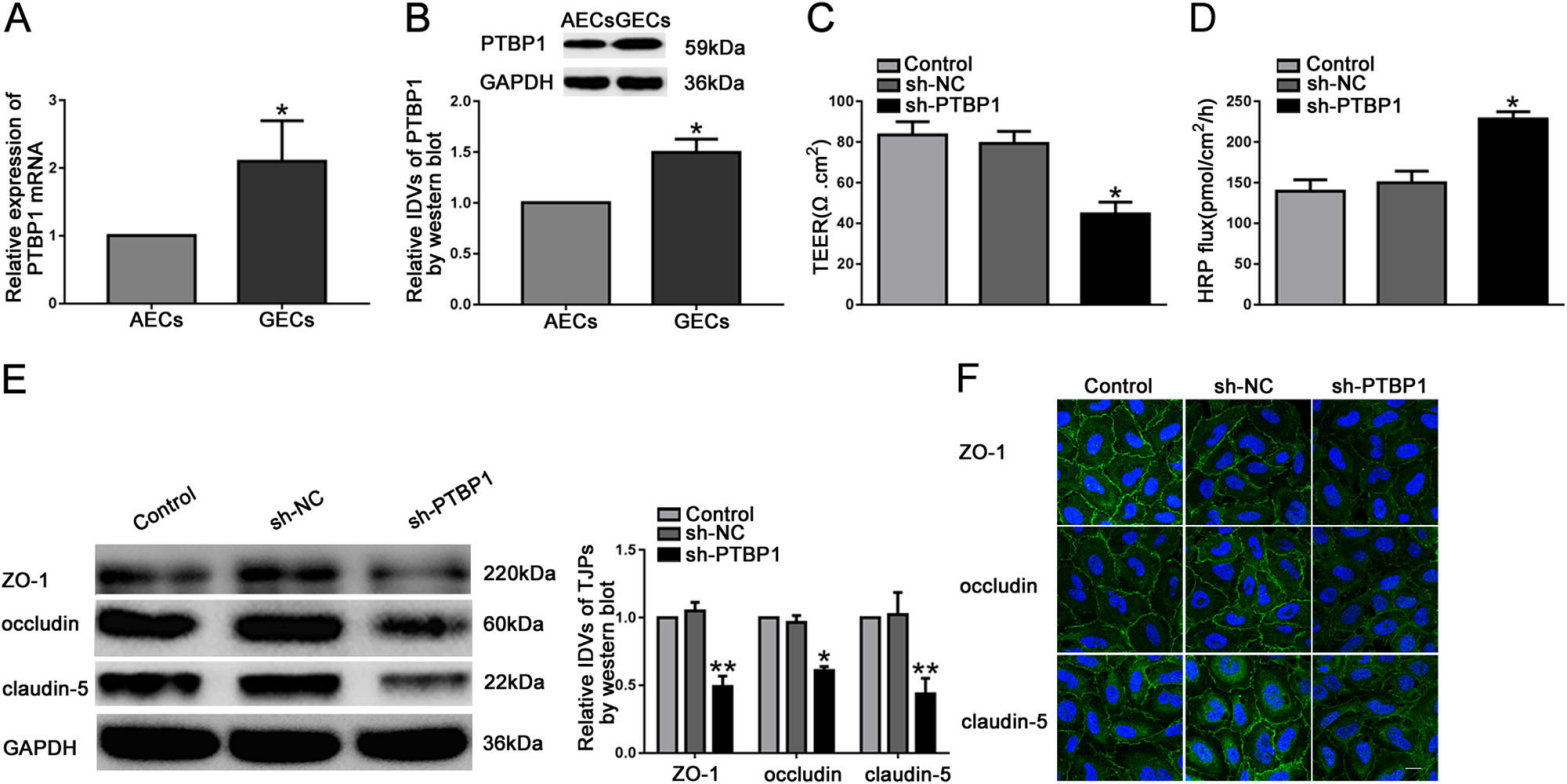
Knockdown of PTBP1 increased BTB permeability in vitro and reduced the expression levels of tight junction-related proteins. **a** Relative PTBP1 expression in AECs and GECs determined by qRT-PCR. Data represent mean ± SD (*n* = 5, each). *P *<* 0.05 versus AECs group. **b** Relative PTBP1 expression in AECs and GECs determined by western blot. Data represent mean ± SD (*n* = 3, each). *P < 0.05 versus AECs group. (C and D) Effects of PTBP1 knockdown on TEER values (**c**) and HRP flux (**d**). **e** Effects of PTBP1 knockdown on ZO-1, occludin, and claudin-5 expression levels determined by western blot. Data represent mean ± SD (*n* = 3, each). *P *<* 0.05, **P < 0.01 versus sh-NC group. **f** Effects of PTBP1 knockdown on ZO-1, occludin, and claudin-5 expression levels and distribution determined by immunofluorescence staining (*n* = 3, each). ZO-1, occludin, and claudin-5 (green) were labeled with secondary antibody against anti-ZO-1, anti-occludin, and anti-claudin-5 antibody, respectively, and nuclei (blue) were labeled with DAPI. Scale bar represents 20 μm.

QRT-PCR and western blot were used to detect the expression of PTBP1. As shown in Fig. 1a, b, PTBP1 was markedly upregulated in GECs. To further validate the effect of PTBP1 on BTB permeability, we transfected ECs with knockdown of PTBP1. In order to assess the integrity and permeability of BTB, we measured the TEER value and HRP flux. There was no significant difference between the knockdown of PTBP1 negative control (sh-NC) group and the control group. Compared with the sh-NC group, the TEER value was evidently decreased and the HRP flux was obviously increased in the knockdown of PTBP1 (sh-PTBP1) group (Fig. 1c, d). Meanwhile, the effects of PTBP1 knockdown on the expression levels of ZO-1, occludin and claudin-5 were shown in Fig. 1e. Compared with the sh-NC group, the expression levels of ZO-1, occludin and claudin-5 significantly decreased in the sh-PTBP1 group.

Furthermore, IF assays confirmed that knockdown of PTBP1 reduced the expression levels of ZO-1, occludin and claudin-5, with discontinuous distribution on the boundaries of GECs (Fig. 1f).

### CircRNA_001160, but not linear ANKRD17, was upregulated in GECs and regulated the expression levels of tight junction-related proteins

**Fig. 2:**
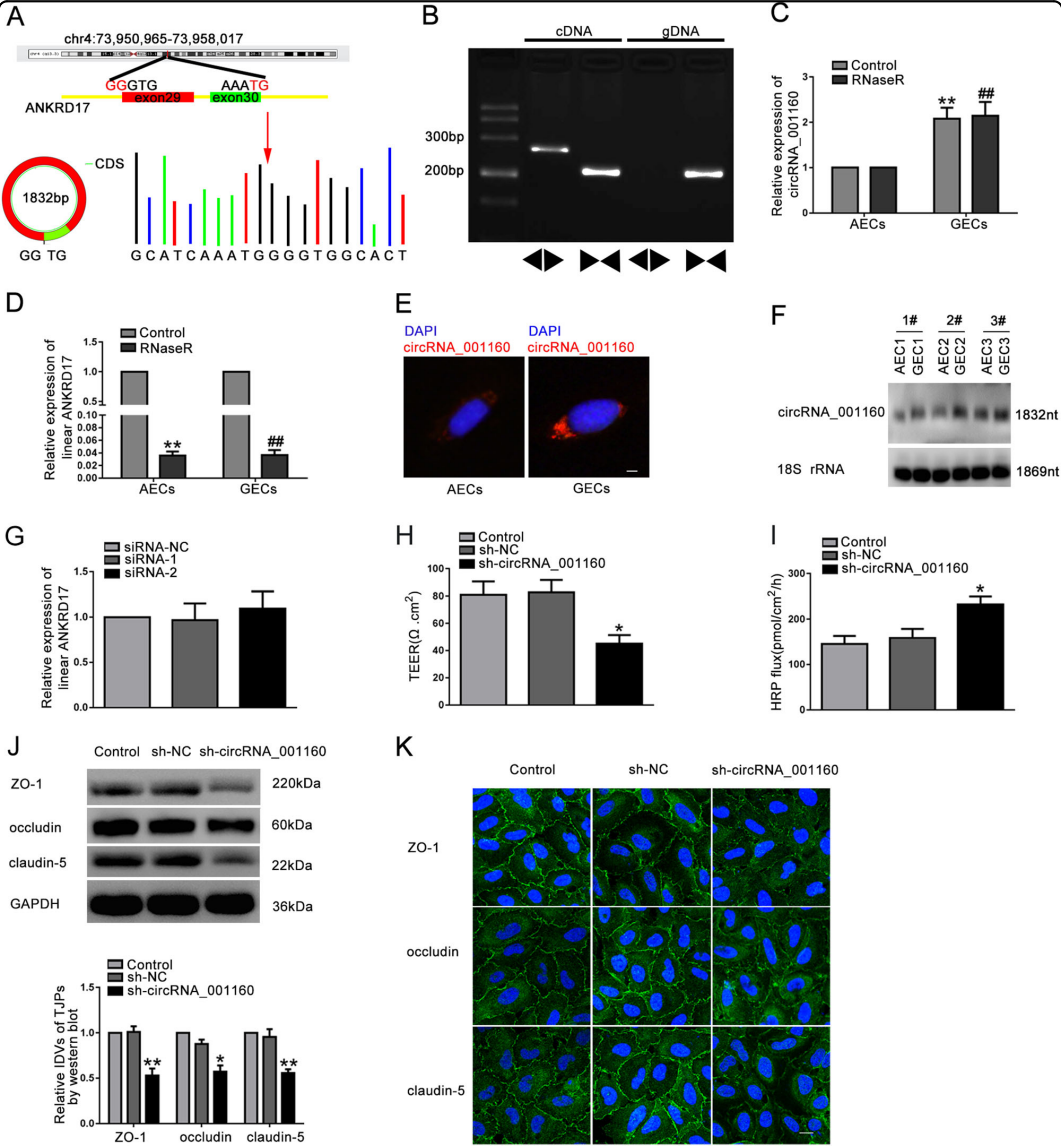
Knockdown of circRNA_001160 increased BTB permeability in vitro and reduced the expression levels of tight junction-related proteins. **a** A schematic representation of how circRNA_001160 arosed from the ANKRD17 gene as determined by scanning ANKRD17 genomic DNA and circBase. Sanger sequencing validated the sequence on the junction sites of circRNA_001160. The red arrow indicated the head-to-tail splicing sites of circRNA_001160. **b**The existence of circRNA_001160 in GECs. **c** The expression of circRNA_001160 in GECs with RNase R treatment. Data represent mean ± SD (*n* = 5, each group). **P < 0.01 versus control group in AECs; ^##^*P* < 0.01 versus RNase R group in AECs. **d** The expression of linear ANKRD17 in GECs with RNase R treatment. Data represent mean ± SD (*n* = 5, each group). **P < 0.01 versus control group in AECs; ^##^*P* < 0.01 versus control group in GECs. **e** The expression and location of circRNA_001160 in AECs and GECs were detected by FISH. (red, circRNA_001160; blue, DAPI nuclear staining). Scale bar represents 20 μm. **f** Relative circRNA_001160 expression in AECs and GECs was detected by northern blotting. **g** The effect of circRNA_001160 knockdown on linear ANKRD17 expression level was detected by qRT-PCR. Data represent mean ± SD (*n* = 5, each group). (H and I) Effects of circRNA_001160 knockdown on TEER values (**h**) and HRP flux (**i**). **j** Effects of circRNA_001160 knockdown on ZO-1, occludin, and claudin-5 expression levels determined by western blot. Data represent mean ± SD (*n* = 3, each). *P *<* 0.05, **P < 0.01 versus sh-NC group. (**k**) Effects of circRNA_001160 knockdown on ZO-1, occludin, and claudin-5 expression levels and distribution determined by immunofluorescence staining (*n* = 3, each). ZO-1, occludin, and claudin-5 (green) were labeled with secondary antibody against anti-ZO-1, anti-occludin, and anti-claudin-5 antibody, respectively, and nuclei (blue) were labeled with DAPI. Scale bar represents 20 μm.

By comparing the genomic DNA sequence of ANKRD17 and circRNA_001160, the composition of circRNA_001160 was shown in Fig. 2a. The sanger sequencing experiment was used to validate the sequence on the junction sites of circRNA_001160. The junction sites of circRNA_001160 are GCATCAAATG and GGGTGGCACT. Divergent primer detected the presence of circRNA_001160 in cDNA and no circRNA_001160 was detected in the genomic DNA (gDNA) (Fig. 2b). RNase R, an RNA exonuclease that degrades linear RNA but does not degrade the circular form, was used to confirm circular RNA. As shown in Fig. 2c, d, circRNA_001160 was resistant to RNase R treatment, whereas linear ANKRD17 treating with RNase R was signifificantly reduced. Notably, there was no significant difference in the expression of linear ANKRD17 (circRNA_001160 parental gene) between the AECs and GECs group. To investigate the expression and location of circRNA_001160, FISH and northern blot were used to detect the expression of circRNA_001160. Results confirmed that circRNA_001160 was located in the cytoplasm and was conspicuously elevated in GECs (Fig. 2e, f). We transfected two circRNA_001160 junction shRNAs in order to determine the effect of circRNA_001160 on the permeability of BTB. These plasmids of shRNAs could specifically knockdown the circRNA_001160 expression level, without affecting the expression of linear ANKRD17 (Fig. S2b, Fig. 2g). After knockdown of circRNA_001160, the TEER value was evidently decreased and the HRP flux was obviously increased (Fig. 2h, i). Knockdown of circRNA_001160 reduced the expression levels of ZO-1, occludin, and claudin-5 (Fig. 2j). IF assays also confirmed that knockdown of circRNA_001160 reduced the expression levels of tight junction-related proteins, and destoryed the continuous distribution of tight junction-related proteins in GECs (Fig. 2k).

### PTBP1 promoted the expression of circRNA_001160

**Fig. 3:**
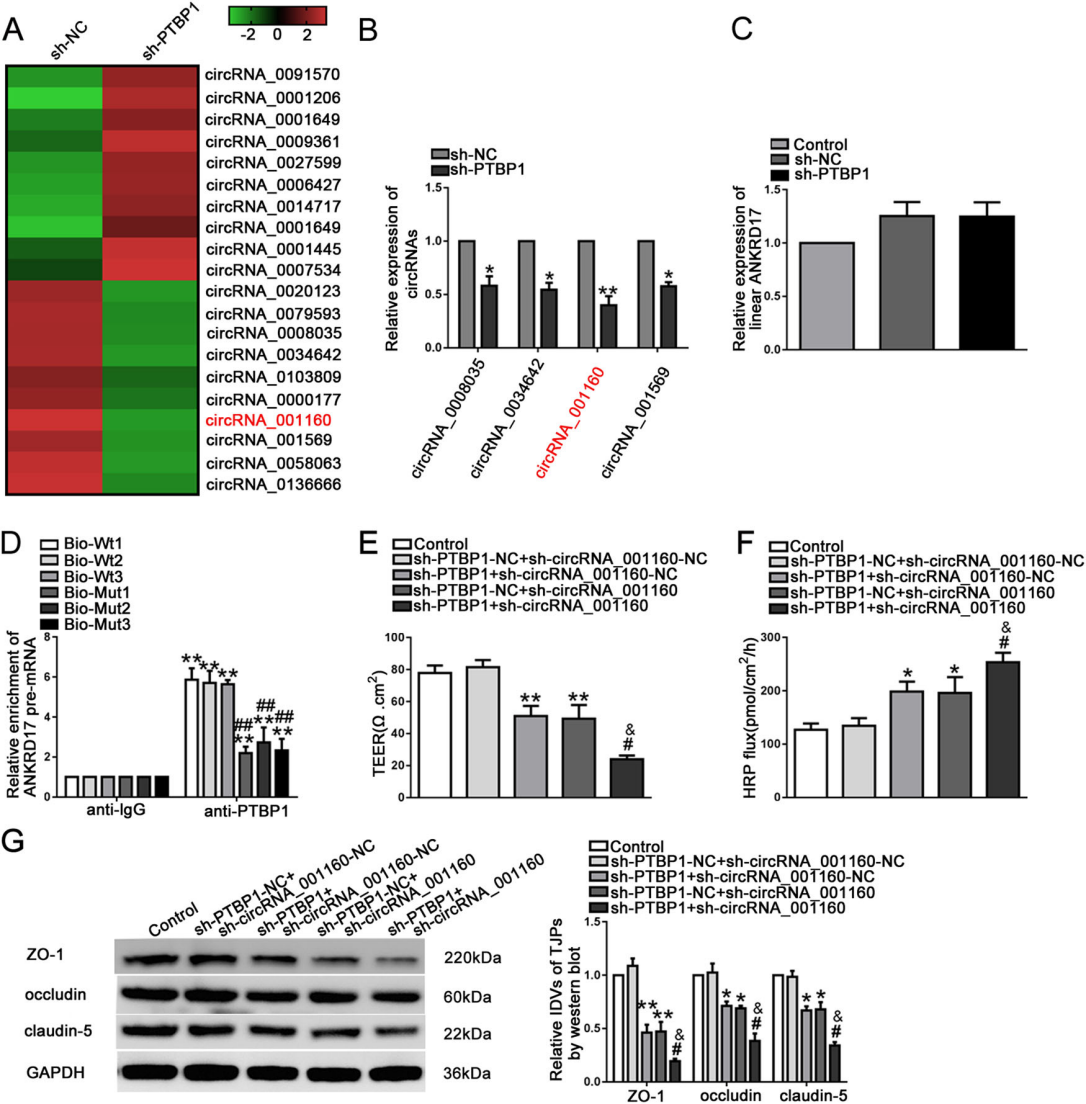
PTBP1 regulated the BTB permeability via promoting the expression of circRNA_001160 in GECs. **a** CircRNA microarray analysis of total RNAs isolated from sh-NC and sh-PTBP1 cells. Red indicates high relative expression and green indicates low relative expression. **b** Relative expression levels of circRNA_0008035, circRNA_0034642, circRNA_001160, and circRNA_001569 were detected by qRT-PCR. Data represent mean ± SD (*n* = 5, each group). *P *<* 0.05, **P *<* 0.01 versus sh-NC group. **c** The effect of PTBP1 knockdown on linear ANKRD17 expression level was detected by qRT-PCR. Data represent mean ± SD (*n* = 5, each group). **d** The relative enrichment of ANKRD17 pre-mRNA in anti-IgG and anti-PTBP1 was detected by a nascent RIP assay. Data represent mean ± SD (*n* = 3, each group). **P < 0.01 versus anti-IgG group; ^##^*P* < 0.01 versus Bio-Wt1-3 in anti-PTBP1 group. **e**, **f** The co-effects of PTBP1 and circRNA_001160 knockdown on TEER values (**e**) and HRP flux (**f**). **g** The co-effects of PTBP1 and circRNA_001160 knockdown on ZO-1, occludin, and claudin-5 expression levels determined by western blot. Data represent mean ± SD (*n* = 3, each). *P < 0.05, **P < 0.01 versus sh-PTBP1-NC + sh-circRNA_001160-NC group; ^#^*P* < 0.05 versus sh-PTBP1 + sh-circRNA_001160-NC group; ^&^*P* < 0.05 versus sh-PTBP1-NC + sh-circRNA_001160 group.

In order to further clarify the mechanism by which PTBP1 regulated BTB permeability, after stable knockdown of PTBP1, we performed a circRNA microarray to assess the expression levels of circRNAs in GECs. As shown in Fig. 3a, circRNA_0008035, circRNA_0034642, circRNA_001160, and circRNA_001569 were the four circRNAs with the most differential expression. QRT-PCR analysis also showed a significant decrease in the expression levels of the four circRNAs (Fig. 3b). In addition, there was no significant change in the expression of linear ANKRD17 between the sh-NC and sh-PTBP group (Fig. 3c). Importantly, the result of Nascent RIP assay was shown in Fig. 3d. Compared with the IgG co-precipitation group, the enrichment of ANKRD17 pre-mRNA in the PTBP1 co-precipitation group was significantly increased. In the PTBP1 co-precipitation group, after the binding sites of PTBP1 and ANKRD17 pre-mRNA were mutated, we found a reduction in the enriched ANKRD17 pre-mRNA. This result further revealed the interaction between PTBP1 and the pre-mRNA of the ANKRD17 gene which formed circRNA_001160. Those results indicated that PTBP1 promoted the expression of circRNA_001160, but had no effect on the expression of linear ANKRD17. To further expore the function of PTBP1 and circRNA_001160, ECs stably expressing sh-PTBP1 and sh-circRNA_001160 were established. Double knockdown of PTBP1 and circRNA_001160 could significantly increase BTB permeability (Fig. 3e–g). These results suggested that PTBP1 regulated BTB permeability by promoting the expression of circRNA_001160.

### MiR-195-5p was downregulated in GECs and regulated BTB permeability

**Fig. 4:**
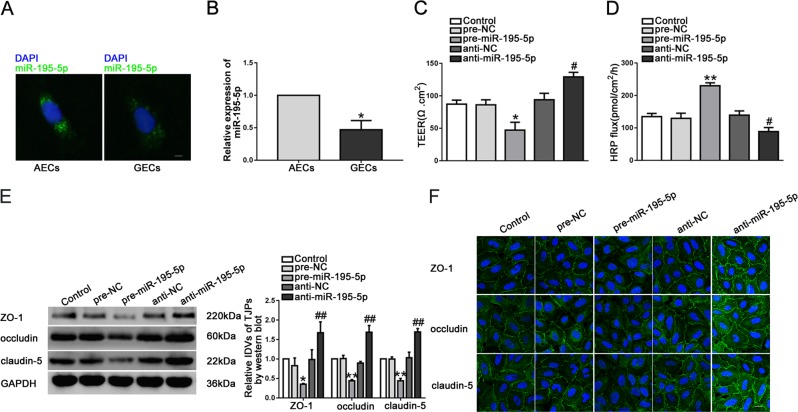
Overexpression of miR-195-5p increased in vitro BTB permeability and reduced the expression levels of tight junction-related proteins. **a** The expression and location of miR-195-5p in AECs and GECs were detected by FISH. (green, miR-195-5p; blue, DAPI nuclear staining). Scale bar represents 20 μm. **b** Relative miR-195-5p expression in AECs and GECs was detected by qRT-PCR. Data represent mean ± SD (*n* = 5, each group). *P < 0.05 versus AECs group. **c**, **d** Effects of miR-195-5p on TEER values (**c**) and HRP flux (**d**). **e** Effects of miR-195-5p on ZO-1, occludin, and claudin-5 expression levels determined by western blot. Data represent mean ± SD (*n* = 3, each). *P *<* 0.05, **P *<* 0.01 versus pre**-**NC group; ^##^*P* *<* 0.01 versus anti-NC group. **f** Effects of miR-195-5p on ZO-1, occludin, and claudin-5 expression levels and distribution determined by immunofluorescence staining (*n* = 3, each). ZO-1, occludin, and claudin-5 (green) were labeled with secondary antibody against anti-ZO-1, anti-occludin, and anti-claudin-5 antibody, respectively, and nuclei (blue) were labeled with DAPI. Scale bar represents 20 μm.

FISH and qRT-PCR were conducted to reveal the expression and location of miR-195-5p. MiR-195-5p was predominately located in the cytoplasm and was downregulated in GECs (Fig. 4a, b). To further investigate the function of miR-195-5p, plasmids with overexpressed and knockdown of miR-195-5p were transfected into ECs. In the pre-miR-195-5p group, the TEER value was evidently decreased, and the HRP flux was obviously increased (Fig. 4c, d). Western blot and IF assays results demonstrated that the expression levels of tight junction-related proteins were significantly downregulated in pre-miR-195-5p group (Fig. 4e, f). The above results indicated that overregulated of miR-195-5p increased BTB permeability.

### CircRNA_001160 negatively regulated and interacted with miR-195-5p

**Fig. 5:**
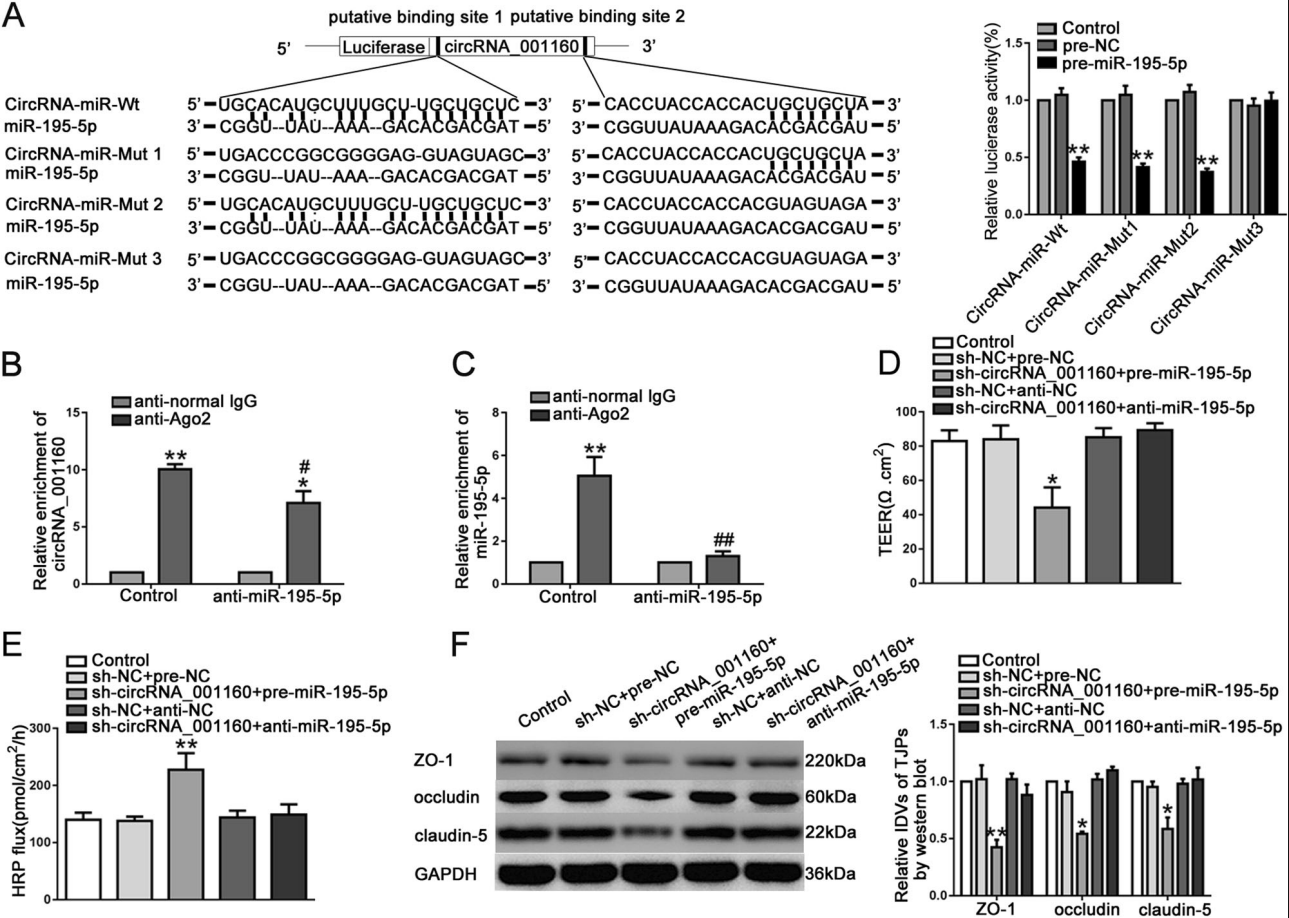
MiR-195-5p targetd circRNA_001160 and reversed the effects of circRNA_001160 on BTB permeability in vitro. **a** Dual-luciferase reporter assays were performed to determine the binding sites of circRNA_001160 and miR-195-5p in HEK293T cells. Data represent mean ± SD (*n* = 5, each). **P *<* 0.01 versus pre-NC group. **b**, **c** MiR-195-5p was identified in circRNA_001160-RISC complex. Relative expression of circRNA_001160 and miR-195-5p were measured using qRT-PCR. Data represent mean ± SD (*n* = 5, each group). *P *<* 0.05, **P *<* 0.01 versus anti-normal IgG group; ^#^*P* *<* 0.05, ^##^*P* < 0.01 versus anti-Ago2 in control group. **d**, **e** The co-effects of circRNA_001160 and miR-195-5p on TEER values (**d**) and HRP flux (**e**). **f** The co-effects of circRNA_001160 and miR-195-5p on ZO-1, occludin, and claudin-5 expression levels determined by western blot. Data represent mean ± SD (*n* = 3, each). *P *<* 0.05, **P < 0.01 versus sh-NC + pre-NC group.

MiRNA microarray analysis was used to screen the circRNA_001160-related miRNAs. We found that miR-195-5p had the highest relative expression after downregulation of circRNA_001160 (Fig. S3a). We applied dual luciferase reporter assay to confirm the existence of targeted binding sites between circRNA_001160 and miR-195-5p. As shown in Fig. 5a, luciferase activity was decreased in HEK293T cells co-transfected with pre-miR-195-5p and circRNA_001160-Wt, pre-miR-195-5p and circRNA_001160-Mut1, pre-miR-195-5p and circRNA_001160-Mut2. However, luciferase activity in HEK293T cells co-transfected with pre-miR-195-5p and circRNA_001160-Mut3 was restored to the control level, suggesting that miR-195-5p bound to circRNA_001160 in a sequence specific manner. Furthermore, RIP experimental results showed that circRNA_001160 and miR-195-5p in Ago2 co-precipitation group were significantly increased (Fig. 5b, c), suggesting that circRNA_001160 and miR-195-5p existed simultaneously in RNA-induced silencing complex (RISC). After knockdown of circRNA_001160, the expression of miR-195-5p was significantly increased. Overexpression of miR-195-5p resulted a significant decrease in the expression of circRNA_001160 (Additional file 1: Fig. S2f, g). These results suggested that circRNA_001160 reduced the expression of miR-195-5p in RISC, and there may be a mutual inhibitory feedback loop between circRNA_001160 and miR-195-5p. Having confirmed that circRNA_001160 bound to miR-195-5p, we further investigated whether miR-195-5p was involved in circRNA_001160-mediated BTB permeability. As shown in Fig. 5d–f, silencing of miR-195-5p significantly reversed the increase of BTB permeability caused by circRNA_001160 knockdown.

### ETV1 was upregulated in GECs and directly bound to the promoter regions of tight junction-related proteins

**Fig. 6:**
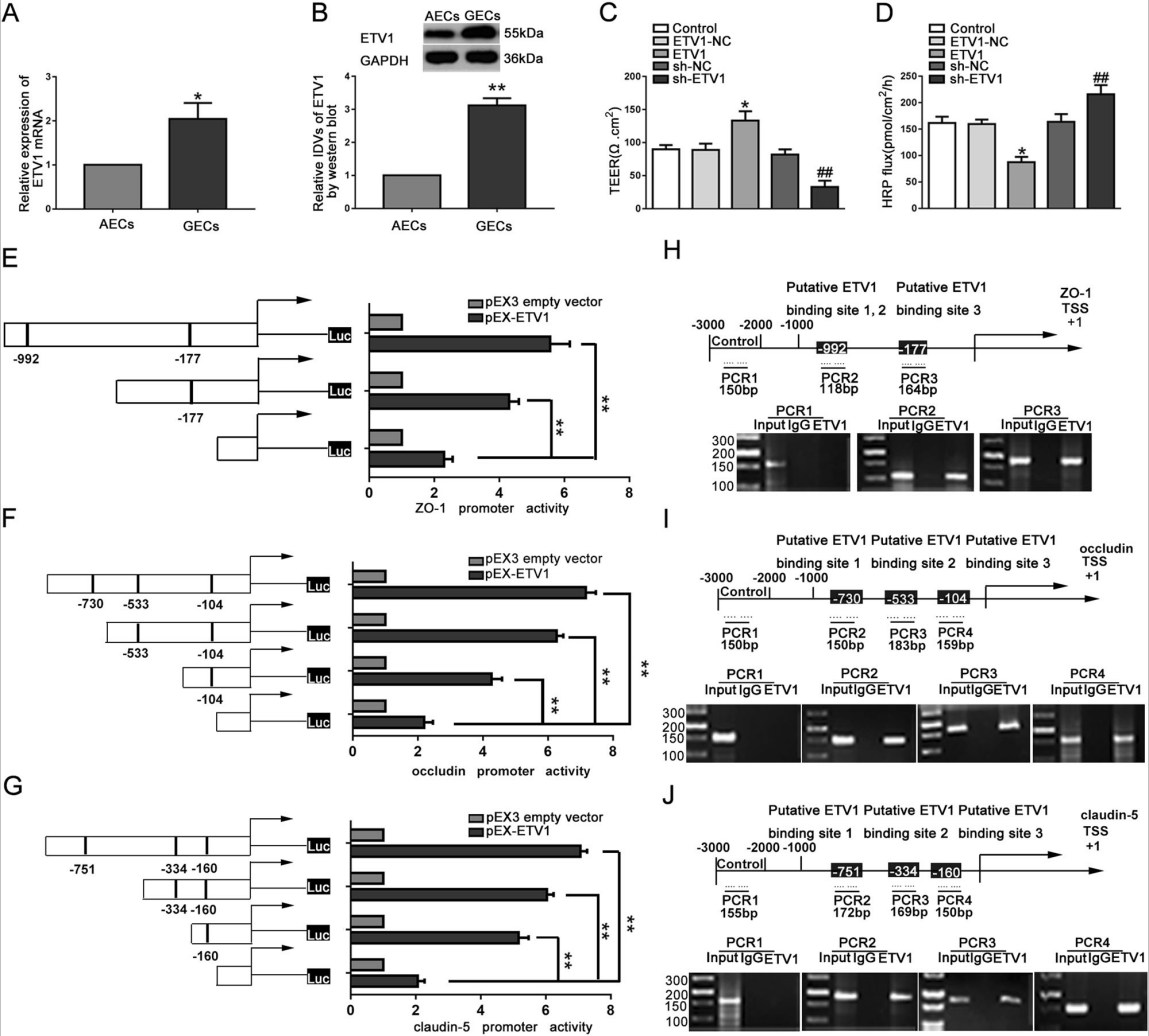
Knockdown of ETV1 increased the BTB permeability and inhibited the expression levels of tight junction-related proteins at the transcriptional level. **a** Relative ETV1 expression in AECs and GECs determined by qRT-PCR. Data represent mean ± SD (*n* = 5, each). *P < 0.05 versus AECs group. (**b**) Relative ETV1 expression in AECs and GECs determined by western blot. Data represent mean ± SD (*n* = 4, each). **P < 0.01 versus AECs group. (C and D) Effects of ETV1 on TEER values (**c**) and HRP flux (**d**). **e**–**g** Schematic depiction of the different reporter plasmids and relative luciferase activity: ZO-1 (**e**), occludin (**f**), and claudin-5 (**g**) were shown. Data represent mean ± SD (*n* = 5, each). **P < 0.01. **h**–**j** Schematic representation of the human ZO-1 (**h**), occludin (**i**), and claudin-5 (**j**) promoter region 3,000 bp upstream of the transcription start sites (TSSs), which were designated as +1. ChIP PCR products for putative ETV1 binding sites and an upstream region not expected to associate with ETV1 were depicted with bold lines. Dashed arrows represent the primers used for each PCR. GECs were used to conduct ChIP assays. PCR was conducted with the resulting precipitated DNA. Images are representative of independent experiments.

QRT-PCR and western blot showed that ETV1 was significantly upregulated in the GECs group (Fig. 6a, b). As shown in Fig. 6c, d, in the ETV1 group, the TEER value was evidently increased, and the HRP flux was obviously decreased. The western blot showed that the expression levels of tight junction-related proteins were significantly increased (Fig. S4b). IF assays showed a high fluorescence intensity and cell boundary enrichment of ZO-1, occludin and claudin-5 in the ETV1 group (Fig. S4c). The results indicated that ETV1 overexpression inhibited BTB permeability by upregulating the expression levels of tight junction-related proteins.

Using the JASPAR CORE database, a total of eight potential binding sites for ETV1 were found in the promoter regions of tight junction-related proteins. The predicted ETV1 binding sites were 177 bp and 992 bp upstream of the ZO-1 transcription initiation site; 104 bp, 553 bp, and 730 bp upstream of the occludin transcription initiation site; and 160 bp, 334 bp and 751 bp upstream of the claudin-5 transcription initiation site. To test whether ETV1 promoted tight promoter activity in tight junction-related proteins in GECs, a series of constructs were designed and the dual-luciferase reporter assays were performed. The results confirmed that ETV1 activated the promoter region activities of tight junction-related proteins (Fig. 6e–g).

Finally, CHIP assays were used to detect whether ETV1 bound to the promoter regions of ZO-1, occludin and claudin-5. As shown in Fig. 6h–j, ETV1 connected with the predicted binding sites of ZO-1, occludin and claudin-5, but did not interact with the control region. The all results indicated that the ETV1 response elements were located in the promoter region of ZO-1, occludin and claudin-5, respectively, and promoted the activity of the promoter regions.

### MiR-195-5p targeted ETV1 3'-UTR and impaired its expression

**Fig. 7:**
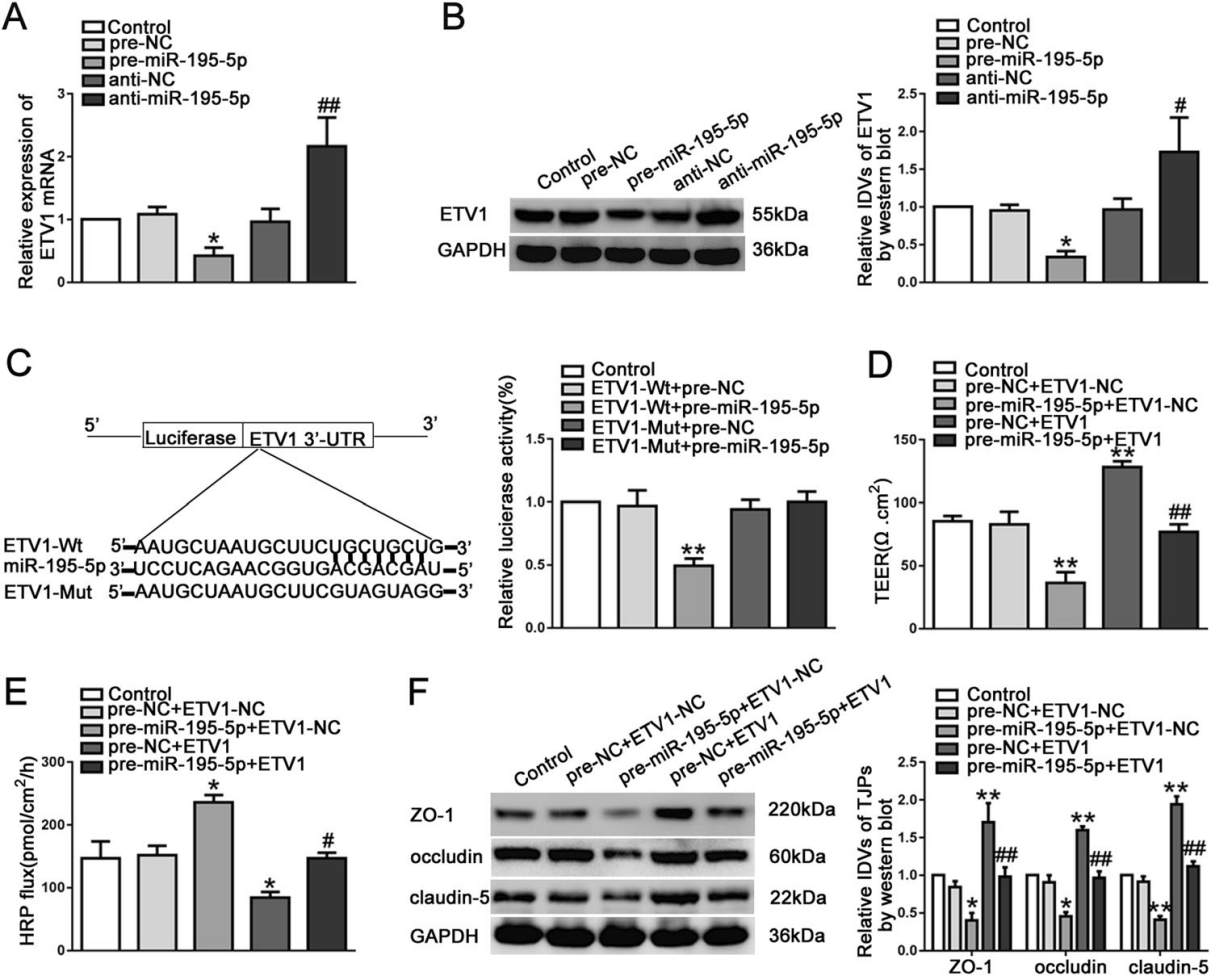
ETV1 was a target of miR-195-5p and was involved in miR-195-5p-mediated BTB permeability in GECs. **a**, **b** The mRNA and protein expressions of ETV1 were regulated by miR-195-5p. Data represent mean ± SD (*n* = 5, each group). *P < 0.05 versus pre-NC group; ^#^*P* < 0.05, ^##^ *P* < 0.01 versus anti-NC group. (**c**) Dual-luciferase reporter assay was performed to determine the binding site of miR-195-5p and ETV1 3′-UTR in HEK293T cells. Data represent mean ± SD (n = 5, each). **P *<* 0.01 versus ETV1-Wt + pre-NC group. **d**, **e** The co-effects of miR-195-5p and ETV1 on TEER values (**d**) and HRP flux (**e**). **f** The co-effects of miR-195-5p and ETV1 on ZO-1, occludin, and claudin-5 expression levels determined by western blot. Data represent mean ± SD (*n* = 3, each). *P < 0.05, **P < 0.01 versus pre-NC + ETV1-NC group; ^##^*P* < 0.01 versus pre-miR-195-5p + ETV1-NC group.

First of all, the mRNA and protein expression levels of ETV1 were examined after changing the expression of miR-195-5p. The mRNA and protein expression levels of ETV1 were significantly decreased in the pre-miR-195-5p group (Fig. 7a, b). Subsequently, the dual luciferase reporter assay showed that co-transfected ETV1-Wt and pre-miR-195-5p group had significantly reduced luciferase activity, while the luciferase activity was no statistically significant in co-transfected ETV1-Mut and pre-miR-195-5p group (Fig. 7c). These results confirmed that miR-195-5p regulated the expression of ETV1 by binding to ETV1 3'-UTR, and ETV1 was a direct target of miR-195-5p. To further investigate whether ETV1 was involved in miR-195-5p-mediated BTB permeability, we established ECs with pre-miR-195-5p and ETV1 plasmids. As shown in Fig. 7d–f, ETV1 overexpression attenuated the decrease in TEER value, the increase in HRP flux and the increase in the expression of tight junction-related proteins induced by miR-195-5p overexpression. The results corroborated that ETV1 participated in miR-195-5p-mediated BTB permeability and overexpression of miR-195-5p attenuated ETV1's regulation of BTB permeability by targeting ETV1 3'-UTR.

### Sh-PTBP1, sh-circRNA_001160, pre-miR-195-5p promoted transmembrane transport of Dox in BTB model and enhanced the anti-glioma effect

**Fig. 8:**
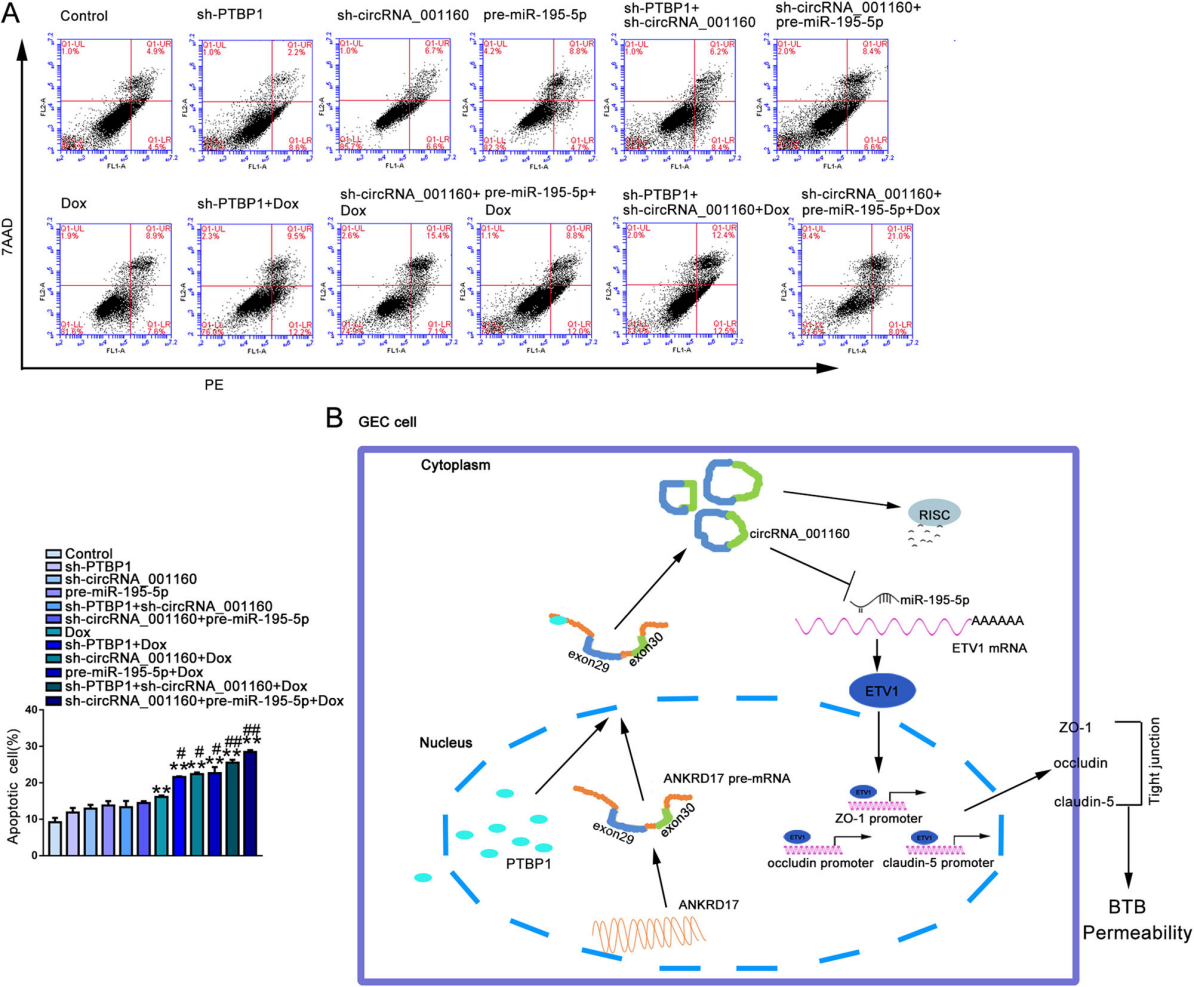
PTBP1, circRNA_001160, miR-195-5p enhanced the effect of Dox in promoting apoptosis of U87 cells. **a** Apoptosis analysis in U87 cells was evaluated by Annexin V-PE/7-AAD staining. Data represent mean ± SD (*n* *=* 3, each). **P < 0.01 versus control group; ^#^*P* < 0.05, ^##^*P* *<* 0.01 versus Dox group. **b** Schematic diagram of PTBP1/circRNA_001160/miR-195-5p/ETV1 pathway to regulate BTB permeability.

As shown in Fig. 8a, compared with the Dox group, the apoptosis rate of U87 cells was distinctly induced in sh-PTBP1 + Dox group, sh-circRNA_001160 + Dox group, pre-miR-195-5p + Dox group, sh-PTBP1 + sh-circRNA_001160 + Dox group and sh-circRNA_001160 + pre-miR-195-5p + Dox group. The results indicated that PTBP1, circRNA_001160 and miR-195-5p could promote the transmembrane transport of Dox, thereby enhancing the role of Dox against glioma. Finally, this article introduced that the PTBP1/circRNA_001160/miR-195-5p/ETV1 axis regulated the mechanism of BTB permeability in GECs.

## Discussion

This study found that PTBP1 and circRNA_001160 were highly upregulated in GECs. The respective knockdown of PTBP1 and circRNA_001160 decreased the expression levels of tight junction-related proteins and increased the permeability of the BTB. In GECs, miR-195-5p was downregulated and ETV1 was overexpressed. Overexpression of miR-195-5p negatively regulated the expression of ETV1, which could reduce the expression levels of tight junction-related proteins. The combination of sh-PTBP1, sh-circRNA_001160, pre-miR-195-5p with Dox significantly induced the apoptosis of U87 cells.

Current researches showed that there are two main ways in which chemotherapeutic drugs can be transported through the BTB, including paracellular pathway and transcellular pathway^36^. The paracellular pathway refers to the transport of substances from blood vessels through the tight junctions between microvascular ECs into brain tissue^37^. Selectively opening tightly linked cell-by-cell pathways is an important way to increase BTB permeability. When the expression levels of tight junction-related proteins are decreased, the BTB permeability is increased^38^. The TEER value and HRP flux involved in this study are important indicators for evaluating the integrity and permeability of BTB. Downregulation of TEER value and upregulation of HRP flux represent the increasing of BTB permeability^39^.

The abnormal expression of RBPs is closely related to the occurrence of various human diseases. Our study first found that knockdown of PTBP1 significantly increased the permeability of BTB, which proved that PTBP1 had a regulatory effect on vascular ECs. Recently, the regulatory effect of PTBP1 on vascular ECs has attracted researchers' attention. Studies have confirmed that PTBP1 promotes proliferation and migration of vascular ECs by forming complexes with cold-shock domain proteins^40^. In patients with pulmonary hypertension, the upregulation of PTBP1 accelerates glycolysis of endothelial cells (BOECs) in blood growth, leading to abnormal metabolism and proliferation of BOECs^41^.

There is increasing evidence that circRNAs regulate various biological behaviors of ECs^42–44^. For the first time, we found that circRNA_001160 knockdown significantly increased BTB permeability. The regulatory effect of circRNAs on vascular ECs can also be seen in cZNF292, which demonstrates the function of promoting angiogenesis in ECs^45^. The knockdown of cZNF609 could increase ECs’ migration and tube formation, reduce retinal vascular loss and inhibit pathological angiogenesis in vivo^46^. CircANRIL induces atherosclerosis by promoting apoptosis and proliferation in human vascular cells and tissues^47^.

There were three binding sites upstream of the region of circRNA_001160 formation on PTBP1 and ANKRD17 pre-mRNA predicted by the application of UCSC and RBPmap database. The experimental data demonstrated that PTBP1 specifically bound to ANKRD17 pre-mRNA. A growing number of studies have indicated that different types of RBPs regulate the expression of circRNAs. The RBP QKI has two potential binding sites upstream and downstream of the circRNA-forming region on the SMARCA5 pre-mRNA. If both upstream or downstream binding sites are mutated, or four sites are simultaneously mutated, the generation of circular RNA is greatly reduced^48^. Eukaryotic initiation factor 4A3 (EIF4A3) binds to the upstream region of the MMP9 mRNA transcript and induces circMMP9 expression^49^. RBP FUS regulates circRNA biogenesis by binding the introns flanking the back-splicing junctions^50^.

MiRNAs are one of the important non-coding RNAs that negatively regulate gene expression at the post-transcriptional level^51^. MiR-195-5p is highly expressed in EC cultures incubated with pre-eclampsia plasma. This state of expression contributes to reduce VEGFA expression and increases anti-angiogenic status in pre-eclampsia^52^. MiR-195-5p also inhibits angiogenesis in liver cancer cells^53^. In the present study, the results showed that miR-195-5p was downregulated in GECs and overexpression of miR-195-5p increased BTB permeability. It was suggested that miR-195-5p could regulate endothelial barrier function.

CircRNAs adsorb miRNAs and act as molecular sponges to regulate the development of multiple malignancies^54–56^. CircRNA-MYLK could directly bind to miR-29a and reduce the inhibition of target VEGFA by miR-29a, thereby promoting the development of bladder cancer^57^. Hsa_circ_0010729 regulates vascular ECs’ proliferation and apoptosis by targeting the miR-186/HIF-1α axis^58^. The results of dual luciferase reporter assays and RIP assays confirmed that miR-195-5p bound to circRNA_001160 and they were involved in the RISC complex. Further, qRT-PCR results showed that miR-195-5p expression was negatively correlated with circRNA_001160 expression, while circRNA_001160 knockdown significantly upregulated the expression of miR-195-5p. At the same time, silencing of miR-195-5p reversed the regulation of circRNA_001160 knockdown on BTB permeability. These results indicated that circRNA_001160 regulated BTB permeability by adsorbing miR-195-5p.

Many studies have confirmed the transcriptional regulation of ETV1 on downstream target proteins. Analysis of DNA sequence data revealed that there are many motifs interacting with Etv1 (DA motif)(G/C/A)GGA(A/T)(G/A) in the promoter region of the mature gene^59^. In prostate cancer, studies have confirmed that ETV1 is capable of binding to the matrix metalloproteinase 7 (MMP-7) gene promoter in vitro and ETV1 stimulates the activity of the MMP-7 promoter^60^. Using the bioinformatics database (targetscan, DBTSS and JASPAR CORE), ETV1 was identified as a direct target of miR-195-5p, and ETV1 was bound to the promoter regions of ZO-1, occludin and claudin-5. We hereby confirmed that ETV1 was highly expressed in GECs and was negatively regulated by miR-195-5p. We further utilized dual-luciferase reporter assays and ChIP assays to show that ETV1 directly bound to the promoter regions of ZO-1, occludin and claudin-5, showing transcriptional promotion. Therefore, ETV1 could regulate the permeability of BTB by a paracellular pathway.

MiRNAs can be used as post-transcriptional gene regulators by targeting the 3'-UTR of target mRNA^61^. We confirmed that miR-195-5p targeted the 3'-UTR of ETV1, and overexpression of miR-195-5p reduced ETV1 mRNA and protein expressions. At the same time, double overexpression of miR-195-5p and ETV1 reversed the pre-miR-195-5p alone on the BTB permeability. The above results confirmed that miR-195-5p regulated the expression of ETV1 at the post-transcriptional level by targeting the 3'-UTR of ETV1 mRNA, thereby regulating BTB permeability.

Doxorubicin (Dox) is a classic anti-neoplasm drug that affects the supercoiling in DNA by inhibiting the activity of the enzyme topoisomerase II^62^. Due to the presence of BTB, the therapeutic effect of Dox on glioma is not obvious^63^. The circular RNA DENND4C could effectively promote the Dox across BTB to induce apoptosis of glioma cells^10^. We found that the combination of sh-PTBP1, sh-circRNA_001160, pre-miR-195-5p with Dox significantly increased the apoptosis of U87 cells. The results indicated the potential value of the combination of genes and drugs in the treatment of glioma.

In summary, PTBP1 promoted the function of circRNA_001160 by regulating the expression of circRNA_001160. Double silencing of PTBP1 and circRNA_001160 could significantly increase BTB permeability. CircRNA_001160 acted as a molecular sponge of miR-195-5p, which had a negative regulatory effect on ETV1. ETV1 promoted the expression levels of tight junction-related proteins at the transcriptional level, and overexpression of ETV1 attenuated BTB permeability. The combined use of PTBP1, circRNA_001160 and miR-195-5p enhanced the role of Dox in promoting apoptosis in glioma cells. The results of this study could provide a new direction for the combined treatment of genes and anti-tumor drugs.

## Supplementary information

Additional file 1: Fig. S1.

Additional file 1: Fig. S2.

Additional file 1: Fig. S3.

Table 4

Table 1

Table 2

Table 3

Additional file 1: Fig. S4.

Supplementary Information

## References

[1] Goncalves CS (2018). WNT6 is a novel oncogenic prognostic biomarker in human glioblastoma. Theranostics.

[2] Buckner JC (2007). Central nervous system tumors. Mayo Clin. Proc..

[3] Zhou W (2017). Targeting glioma stem cell-derived pericytes disrupts the blood-tumor barrier and improves chemotherapeutic efficacy. Cell Stem Cell.

[4] Toyoda K (2013). Initial contact of glioblastoma cells with existing normal brain endothelial cells strengthen the barrier function via fibroblast growth factor 2 secretion: a new in vitro blood-brain barrier model. Cell Mol. Neurobiol..

[5] Gril B (2018). Reactive astrocytic S1P3 signaling modulates the blood-tumor barrier in brain metastases. Nat. Commun..

[6] Arvanitis CD (2018). Mechanisms of enhanced drug delivery in brain metastases with focused ultrasound-induced blood-tumor barrier disruption. Proc. Natl Acad. Sci. USA.

[7] Pardridge WM (1998). CNS drug design based on principles of blood-brain barrier transport. J. Neurochem..

[8] Zhang J (2017). Increasing of blood-brain tumor barrier permeability through transcellular and paracellular pathways by microbubble-enhanced diagnostic ultrasound in a C6 glioma model. Front. Neurosci..

[9] Li Z (2016). Low-dose endothelial monocyte-activating polypeptide-II induces blood-tumor barrier opening via the cAMP/PKA/Rac1 pathway. J. Mol. Neurosci..

[10] Wu P (2019). KHDRBS3 regulates the permeability of blood-tumor barrier via cDENND4C/miR-577 axis. Cell Death Dis..

[11] Glisovic T, Bachorik JL, Yong J, Dreyfuss G (2008). RNA-binding proteins and post-transcriptional gene regulation. Febs. Lett..

[12] Bushell M (2006). Polypyrimidine tract binding protein regulates IRES-mediated gene expression during apoptosis. Mol. Cell.

[13] Zhang L, Yang Z, Huang W, Wu J (2019). H19 potentiates let-7 family expression through reducing PTBP1 binding to their precursors in cholestasis. Cell Death Dis..

[14] David CJ, Chen M, Assanah M, Canoll P, Manley JL (2010). HnRNP proteins controlled by c-Myc deregulate pyruvate kinase mRNA splicing in cancer. Nature.

[15] Bielli P (2018). The splicing factor PTBP1 promotes expression of oncogenic splice variants and predicts poor prognosis in patients with non-muscle-invasive bladder cancer. Clin. Cancer Res..

[16] Cheng C (2018). PTBP1 knockdown overcomes the resistance to vincristine and oxaliplatin in drug-resistant colon cancer cells through regulation of glycolysis. Biomed. Pharmacother.

[17] Wang X (2018). PTBP1 promotes the growth of breast cancer cells through the PTEN/Akt pathway and autophagy. J. Cell Physiol..

[18] He Q (2018). circ-SHKBP1 regulates the angiogenesis of U87 glioma-exposed endothelial cells through miR-544a/FOXP1 and miR-379/FOXP2 pathways. Mol. Ther. Nucleic Acids.

[19] He Z (2019). FUS/circ_002136/miR-138-5p/SOX13 feedback loop regulates angiogenesis in Glioma. J. Exp. Clin. Cancer Res..

[20] Anderson DM (2015). A micropeptide encoded by a putative long noncoding RNA regulates muscle performance. Cell.

[21] Zhang M (2018). A peptide encoded by circular form of LINC-PINT suppresses oncogenic transcriptional elongation in glioblastoma. Nat. Commun..

[22] Vandell AG (2014). Hydrochlorothiazide-induced hyperuricaemia in the pharmacogenomic evaluation of antihypertensive responses study. J. Intern. Med..

[23] Sidor CM, Brain R, Thompson BJ (2013). Mask proteins are cofactors of Yorkie/YAP in the Hippo pathway. Curr. Biol..

[24] Zhou K (2018). Knockdown of long non-coding RNA NEAT1 inhibits glioma cell migration and invasion via modulation of SOX2 targeted by miR-132. Mol. Cancer.

[25] Krol J, Loedige I, Filipowicz W (2010). The widespread regulation of microRNA biogenesis, function and decay. Nat. Rev. Genet..

[26] Toyama K (2018). MicroRNA-mediated therapy modulating blood-brain barrier disruption improves vascular cognitive impairment. Arterioscler. Thromb. Vasc. Biol..

[27] Zhao L, Wang P, Liu Y, Ma J, Xue Y (2015). miR-34c regulates the permeability of blood-tumor barrier via MAZ-mediated expression changes of ZO-1, occludin, and claudin-5. J. Cell Physiol..

[28] Ma J (2014). MiR-181a regulates blood-tumor barrier permeability by targeting Kruppel-like factor 6. J. Cereb. Blood Flow. Metab..

[29] Dai J, Wei R, Zhang P, Kong B (2019). Overexpression of microRNA-195-5p reduces cisplatin resistance and angiogenesis in ovarian cancer by inhibiting the PSAT1-dependent GSK3beta/beta-catenin signaling pathway. J. Transl. Med..

[30] Zhang QQ (2012). MicroRNA-195 plays a tumor-suppressor role in human glioblastoma cells by targeting signaling pathways involved in cellular proliferation and invasion. Neuro. Oncol..

[31] Jomrich G (2018). MK2 and ETV1 are prognostic factors in esophageal adenocarcinomas. J. Cancer.

[32] Hollenhorst PC, McIntosh LP, Graves BJ (2011). Genomic and biochemical insights into the specificity of ETS transcription factors. Annu. Rev. Biochem..

[33] Jeon IS (1995). A variant Ewing's sarcoma translocation (7;22) fuses the EWS gene to the ETS gene ETV1. Oncogene.

[34] Jane-Valbuena J (2010). An oncogenic role for ETV1 in melanoma. Cancer Res..

[35] Li J (2017). miR-17-5p suppresses cell proliferation and invasion by targeting ETV1 in triple-negative breast cancer. Bmc. Cancer.

[36] Xie H, Xue YX, Liu LB, Liu YH, Wang P (2012). Role of RhoA/ROCK signaling in endothelial-monocyte-activating polypeptide II opening of the blood-tumor barrier: role of RhoA/ROCK signaling in EMAP II opening of the BTB. J. Mol. Neurosci..

[37] Li Z (2015). Functions for the cAMP/Epac/Rap1 signaling pathway in low-dose endothelial monocyte-activating polypeptide-II-induced opening of blood-tumor barrier. J. Mol. Neurosci..

[38] Fan L (2011). Increasing of blood-tumor barrier permeability through paracellular pathway by low-frequency ultrasound irradiation in vitro. J. Mol. Neurosci..

[39] Shen S (2018). PIWIL1/piRNA-DQ593109 regulates the permeability of the blood-tumor barrier via the MEG3/miR-330-5p/RUNX3 axis. Mol. Ther. Nucleic Acids.

[40] Masuda K, Abdelmohsen K, Gorospe M (2009). RNA-binding proteins implicated in the hypoxic response. J. Cell Mol. Med..

[41] Caruso P (2017). Identification of MicroRNA-124 as a major regulator of enhanced endothelial cell glycolysis in pulmonary arterial hypertension via PTBP1 (polypyrimidine tract binding protein) and pyruvate kinase M2. Circulation.

[42] Su W, Sun S, Wang F, Shen Y, Yang H (2019). Circular RNA hsa_circ_0055538 regulates the malignant biological behavior of oral squamous cell carcinoma through the p53/Bcl-2/caspase signaling pathway. J. Transl. Med..

[43] Ling L (2019). CircRNAs in exosomes from high glucose-treated glomerular endothelial cells activate mesangial cells. Am. J. Transl. Res..

[44] Meng Q (2019). Circular RNA circSCAF11 accelerates the glioma tumorigenesis through the miR-421/SP1/VEGFA Axis. Mol. Ther. Nucleic Acids.

[45] Boeckel JN (2015). Identification and characterization of hypoxia-regulated endothelial circular RNA. Circ. Res..

[46] Liu C (2017). Silencing of circular RNA-ZNF609 ameliorates vascular endothelial dysfunction. Theranostics.

[47] Holdt LM (2016). Circular non-coding RNA ANRIL modulates ribosomal RNA maturation and atherosclerosis in humans. Nat. Commun..

[48] Conn SJ (2015). The RNA binding protein quaking regulates formation of circRNAs. Cell.

[49] Wang R (2018). EIF4A3-induced circular RNA MMP9 (circMMP9) acts as a sponge of miR-124 and promotes glioblastoma multiforme cell tumorigenesis. Mol. Cancer.

[50] Errichelli L (2017). FUS affects circular RNA expression in murine embryonic stem cell-derived motor neurons. Nat. Commun..

[51] Zhang X (2019). miR498 inhibits the growth and metastasis of liver cancer by targeting ZEB2. Oncol. Rep..

[52] Sandrim VC (2016). Plasma from pre-eclamptic patients induces the expression of the anti-angiogenic miR-195-5p in endothelial cells. J. Cell Mol. Med..

[53] Wang R (2013). MicroRNA-195 suppresses angiogenesis and metastasis of hepatocellular carcinoma by inhibiting the expression of VEGF, VAV2, and CDC42. Hepatology.

[54] Li M (2018). A circular transcript of ncx1 gene mediates ischemic myocardial injury by targeting miR-133a-3p. Theranostics.

[55] Chen, Y. et al. Circ-ASH2L promotes tumor progression by sponging miR-34a to regulate Notch1 in pancreatic ductal adenocarcinoma. *J. Exp. Clin. Cancer Res.* **38**, 466 (2019). 10.1186/s13046-019-1436-0 PMC6852927 31718694

[56] Chen X (2018). PRMT5 circular RNA promotes metastasis of urothelial carcinoma of the bladder through sponging miR-30c to induce epithelial-mesenchymal transition. Clin. Cancer Res..

[57] Zhong Z (2017). Circular RNA MYLK as a competing endogenous RNA promotes bladder cancer progression through modulating VEGFA/VEGFR2 signaling pathway. Cancer Lett..

[58] Dang RY, Liu FL, Li Y (2017). Circular RNA hsa_circ_0010729 regulates vascular endothelial cell proliferation and apoptosis by targeting the miR-186/HIF-1alpha axis. Biochem. Biophys. Res. Commun..

[59] Flames, N. & Hobert, O. Gene regulatory logic of dopamine neuron differentiation. *Nature* **458**, 885–889 (2009). 10.1038/nature07929 PMC2671564 19287374

[60] Shin S, Oh S, An S, Janknecht R (2013). ETS variant 1 regulates matrix metalloproteinase-7 transcription in LNCaP prostate cancer cells. Oncol. Rep..

[61] Ambros V (2004). The functions of animal microRNAs. Nature.

[62] Han J (2016). Rapid emergence and mechanisms of resistance by U87 glioblastoma cells to doxorubicin in an in vitro tumor microfluidic ecology. Proc. Natl Acad. Sci. USA.

[63] Cui, Y., Xu, Q., Chow, P. K., Wang, D. & Wang, C. H. Transferrin-conjugated magnetic silica PLGA nanoparticles loaded with doxorubicin and paclitaxel for brain glioma treatment. *B**iomaterials* **34**, 8511–8520 (2013). 10.1016/j.biomaterials.2013.07.075 23932498

