# Supplementary figures and images for: Biosynthetic CircRNA_001160 induced by PTBP1 regulates the permeability of BTB via the CircRNA_001160/miR-195-5p/ETV1 axis
Source: Cell Death Dis. 2019 Dec 20;10(12):960. doi: 10.1038/s41419-019-2191-z (PMC6925104; doi:10.1038/s41419-019-2191-z)

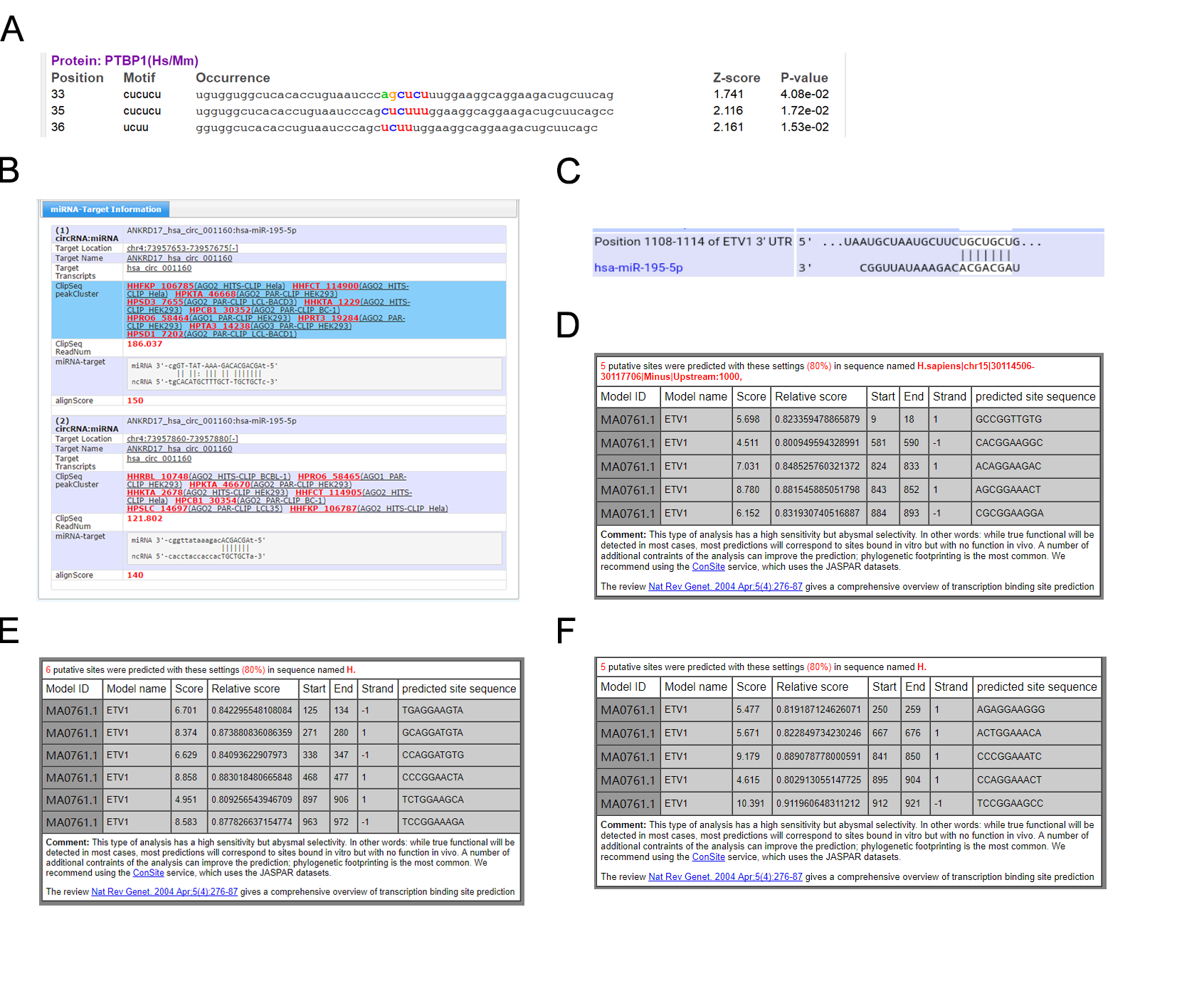

Supplement: Supplementary file 1 — Additional file 1: Fig. S1. [file 41419_2019_2191_MOESM1_ESM.tif]

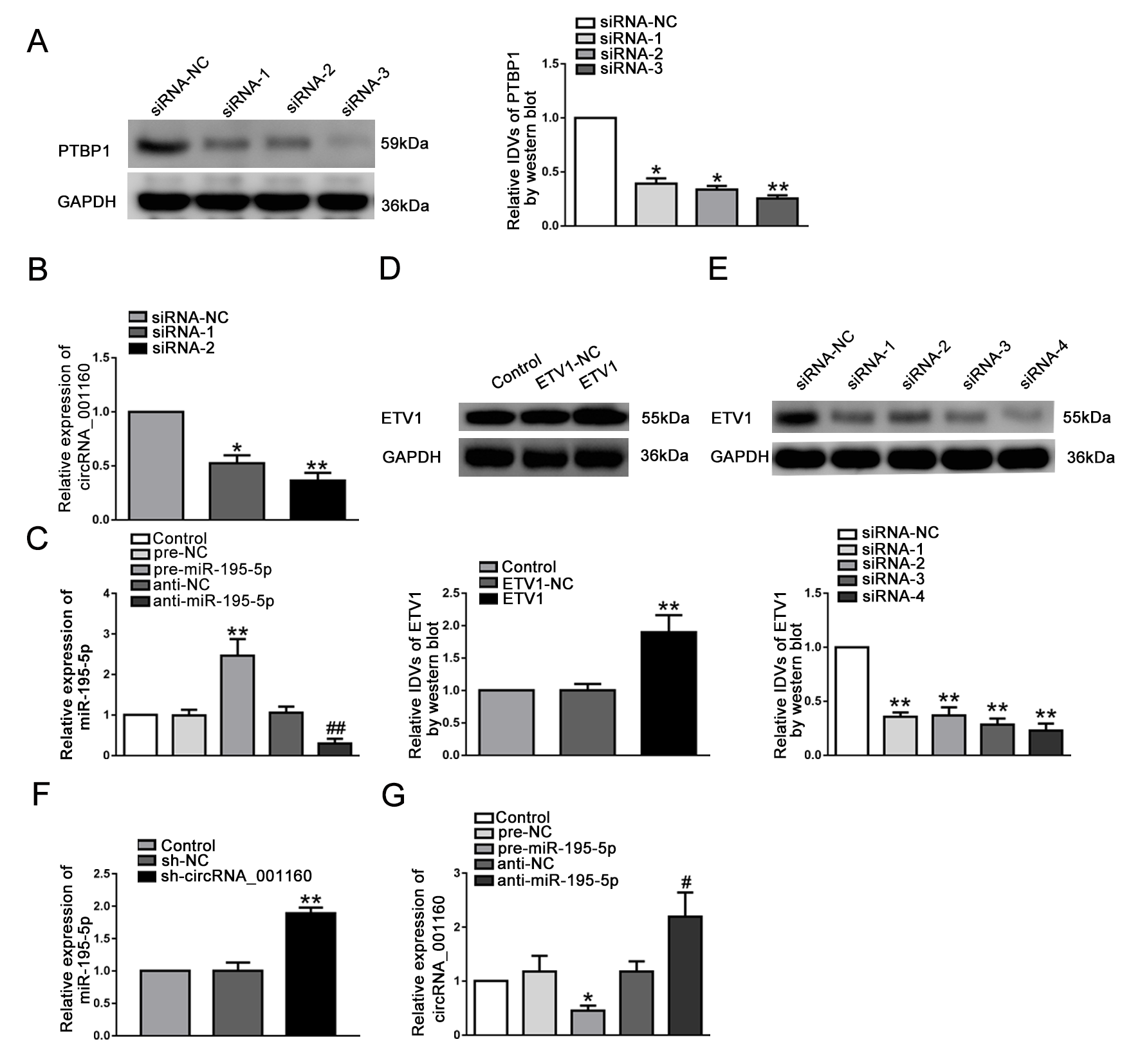

Supplement: Supplementary file 2 — Additional file 1: Fig. S2. [file 41419_2019_2191_MOESM2_ESM.tif]

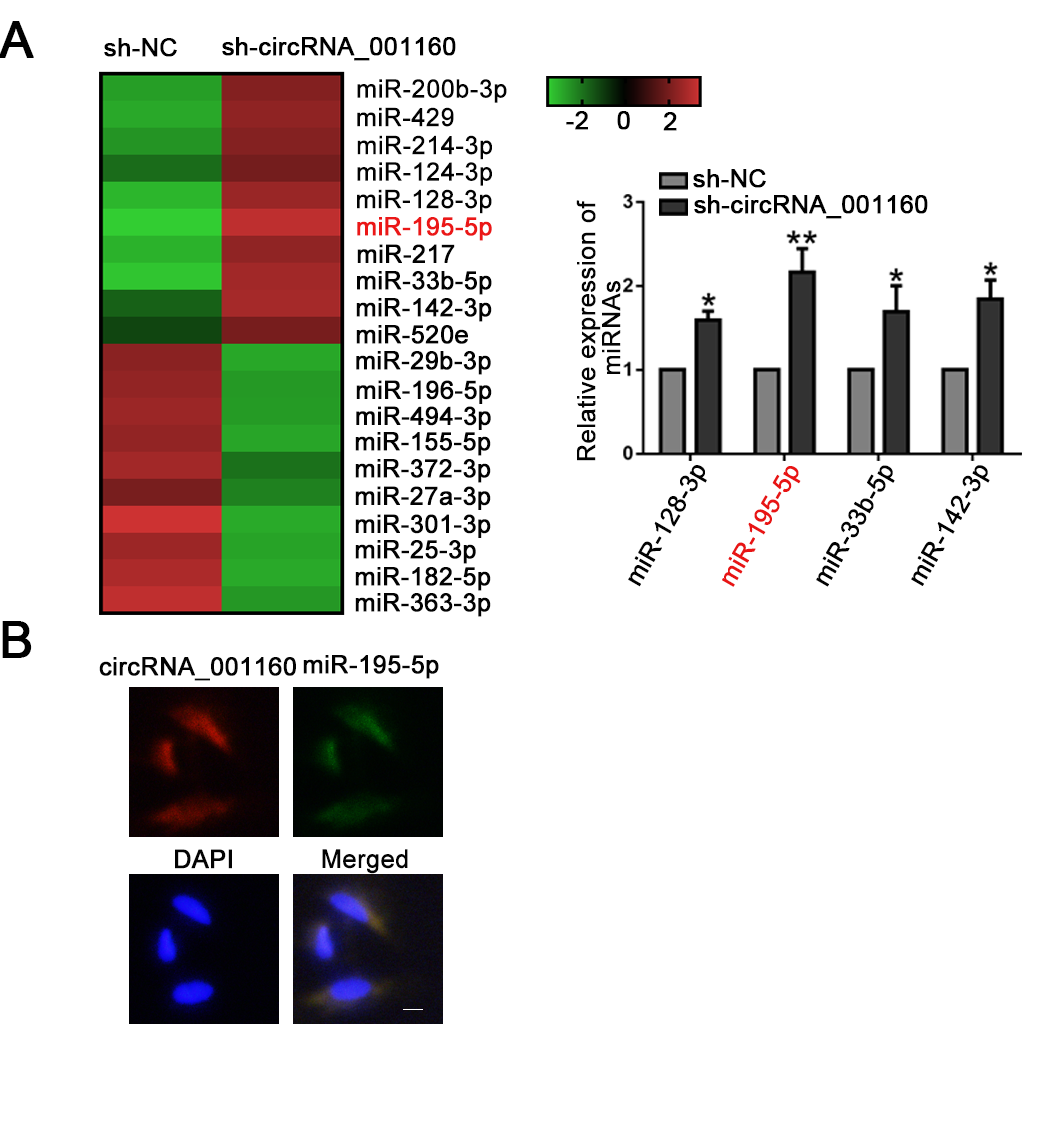

Supplement: Supplementary file 3 — Additional file 1: Fig. S3. [file 41419_2019_2191_MOESM3_ESM.tif]

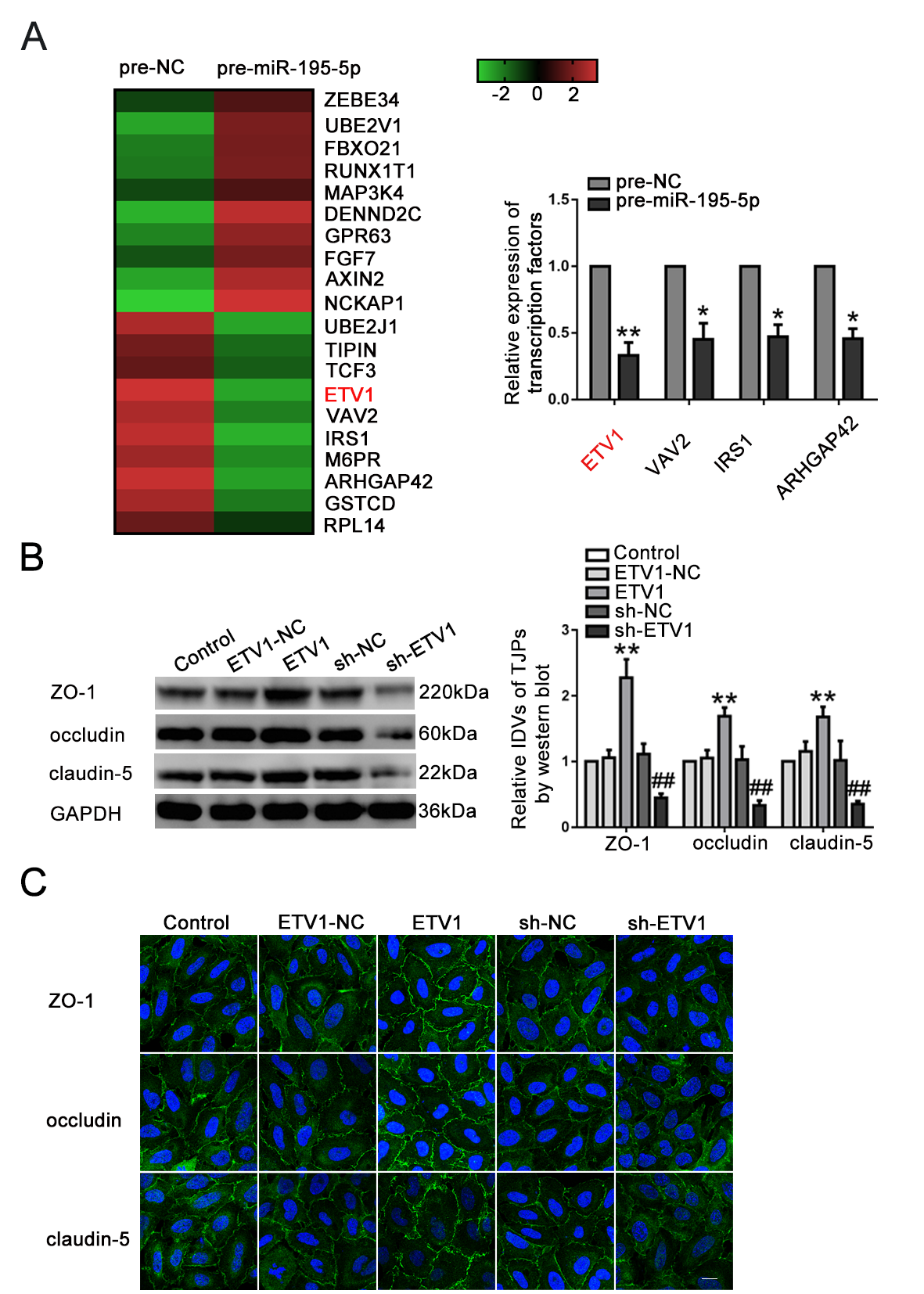

Supplement: Supplementary file 8 — Additional file 1: Fig. S4. [file 41419_2019_2191_MOESM8_ESM.tif]
