# Supplementary material for: Biosynthetic CircRNA_001160 induced by PTBP1 regulates the permeability of BTB via the CircRNA_001160/miR-195-5p/ETV1 axis
Source: Cell Death Dis. 2019 Dec 20;10(12):960. doi: 10.1038/s41419-019-2191-z (PMC6925104; doi:10.1038/s41419-019-2191-z)
Supplement: Supplementary file 4 — Table 4 [file 41419_2019_2191_MOESM4_ESM.docx]

Table 4

Wild-type and mutant plasmid sequences

| Wild-type plasmid sequences | Mutant plasmid sequences |
| --- | --- |
| AUCCCAGCUCUUUGGA | AUCCCCUAGAGUUGGA |
| CCCAGCUCUUUGGAAG | CCCAGAGAGGGGGAAG |
| CCAGCUCUUUGGAA | CCAGCGAGGUGGAA |
