# Supplementary material for: Biosynthetic CircRNA_001160 induced by PTBP1 regulates the permeability of BTB via the CircRNA_001160/miR-195-5p/ETV1 axis
Source: Cell Death Dis. 2019 Dec 20;10(12):960. doi: 10.1038/s41419-019-2191-z (PMC6925104; doi:10.1038/s41419-019-2191-z)
Supplement: Supplementary file 5 — Table 1 [file 41419_2019_2191_MOESM5_ESM.docx]

Table 1

Primers and probes used for qRT-PCR

| Primer or Probe | Gene | Sequence(5’->3’) or Assay ID |
| --- | --- | --- |
| Primer | PTBP1 | F:GCTGCACCTCTCCAACATCC |
|  |  | R:GTCGTGGTTGTGCAGGTCAA |
|  | CircRNA_001160 | F:TCCCCGCTTAGTTTGTTGAC |
|  |  | R:CAATTTCTTTGTCTGGATCCTTG |
|  |  | Probe:FAM + ATCAGCATCAAATGGGGTGGCACTG+BHQ1 |
|  | GAPDH | F:GGACCTGACCTGCCGTCTAG |
|  |  | R:TAGCCCAGGATGCCCTTGAG |
|  |  | Probe:FAM+CCTCCGACGCCTGCTTCACCACCT+Eclipse |
|  | Linear ANKRD17 | F:CCACGGCAAGCAACAACAACAC |
|  |  | R:CCTCCTGGCTGGGCTGGTG |
|  | ANKRD17 pre-mRNA | F:CAGGAGGTCAGATGTACGGA |
|  |  | R:TCAGTGCCACCCCATTTGAT |
|  | CircRNA_001160 convergent primers | F:TCACAACCACGGCAAGCAAC |
|  |  | R:ACCTGCTTCCTGTTCGGAGG |
|  | CircRNA_001160 divergent primers | F:AGATGTACGGACCTGGGGCA |
|  |  | R:TGGTGGTAGGTGCTGATGACC |
|  | ETV1 | F:GACGATGCCAAGGGAAGGAC |
|  |  | R:ACTGGGTCGTGGTACTCCTG |
| Probe | miR-195-5p | 000494(Applied biosystems) |
|  | U6 | 001973(Applied biosystems) |
