# Supplementary material for: Biosynthetic CircRNA_001160 induced by PTBP1 regulates the permeability of BTB via the CircRNA_001160/miR-195-5p/ETV1 axis
Source: Cell Death Dis. 2019 Dec 20;10(12):960. doi: 10.1038/s41419-019-2191-z (PMC6925104; doi:10.1038/s41419-019-2191-z)
Supplement: Supplementary file 6 — Table 2 [file 41419_2019_2191_MOESM6_ESM.docx]

Table 2

Sequences of shRNA template

| Gene | Sequence(5’->3’) | |
| --- | --- | --- |
| PTBP1 | Sense | CCGGAGCAAACGGAAATGACAGCAACTCGAGTTGCTGTCATTTCCGTTTGCTTTTTTG |
|  | Antisense | GATCCAAAAAAGCAAACGGAAATGACAGCAACTCGAGTTGCTGTCATTTCCGTTTGCT |
| CircRNA_001160 | Sense | CACCGCATCAAATGGGGTGGCACTGTTCAAGAGACAGTGCCACCCCATTTGATGCTTTTTTG |
|  | Antisense | GATCCAAAAAAGCATCAAATGGGGTGGCACTGTCTCTTGAACAGTGCCACCCCATTTGATGC |
| ETV1 | Sense | CACCGGGTCGAGGCATGGAATTTATTCAAGAGATAAATTCCATGCCTCGACCTTTTTTG |
|  | Antisense | GATCCAAAAAAGGTCGAGGCATGGAATTTATCTCTTGAATAAATTCCATGCCTCGACCC |
| NC | Sense | CACCGTTCTCCGAACGTGTCACGTCAAGAGATTACGTGACACGTTCGGAGAATTTTTTG |
|  | Antisense | GATCCAAAAAAGTTCTCCGAACGTGTCACGTAATCTCTTGACGTGACACGTTCGGAGAAC |
