# Supplementary material for: Biosynthetic CircRNA_001160 induced by PTBP1 regulates the permeability of BTB via the CircRNA_001160/miR-195-5p/ETV1 axis
Source: Cell Death Dis. 2019 Dec 20;10(12):960. doi: 10.1038/s41419-019-2191-z (PMC6925104; doi:10.1038/s41419-019-2191-z)
Supplement: Supplementary file 7 — Table 3 [file 41419_2019_2191_MOESM7_ESM.docx]

Table 3

Primers used for CHIP experiments

| Gene | Binding site or control | Sequence(5’->3’) | Product size(bp) | Annealing temperature(°C) |
| --- | --- | --- | --- | --- |
| ZO-1 | PCR1 | CCCCAAGTGCTGGGATTAT | 150 | 57.5 |
|  |  | GGAGGTTGCAGTGAGTCCAG |  |  |
|  | PCR2 | GAAAACCATTGTCTAAAGCCTGA | 118 | 53.2 |
|  |  | TGCCAGAAATCCTTAGTAACATCA |  |  |
|  | PCR3 | GGCATGCTCAGTGGGCCG | 164 | 63.7 |
|  |  | CGTCAGCAGCACCCGTGG |  |  |
| occludin | PCR1 | GATTAACCTGACTTCCCCAGTG | 150 | 56.7 |
|  |  | TTTCAGATGATGGCAGCTTT |  |  |
|  | PCR2 | GGGAGAGTCACATCTCTAACCA | 150 | 58.3 |
|  |  | AACTATCTGCATTGAAAGCGAAC |  |  |
|  | PCR3 | CCCTTGGAAACAGAATCCAG | 183 | 54.9 |
|  |  | GCTCTTTGGCCTGAGAAAAA |  |  |
|  | PCR4 | AGGAGTCTTTCGTTGGAGCA | 159 | 59.5 |
|  |  | AGGTCCAGAGGGGACTGTTT |  |  |
| Claudin-5 | PCR1 | TCCACTCTGGATGCTGCTC | 155 | 58.4 |
|  |  | GCCTGGTGCTCTTTCTTGAC |  |  |
|  | PCR2 | GGGTTAGGGGGTAGTAAGAGTGA | 172 | 60.2 |
|  |  | TCTGAGGACCACTGCCTTCT |  |  |
|  | PCR3 | TCTGAGGACCACTGCCTTCT | 169 | 58.7 |
|  |  | GGCTCCAAAACCCACAGC |  |  |
|  | PCR4 | CATGGGGCTGTGGGTTTT | 150 | 58.4 |
|  |  | CCATCCTTGCTGGAGGAG |  |  |
