# Supplementary material for: Biosynthetic CircRNA_001160 induced by PTBP1 regulates the permeability of BTB via the CircRNA_001160/miR-195-5p/ETV1 axis
Source: Cell Death Dis. 2019 Dec 20;10(12):960. doi: 10.1038/s41419-019-2191-z (PMC6925104; doi:10.1038/s41419-019-2191-z)
Supplement: Supplementary file 9 — Supplementary Information [file 41419_2019_2191_MOESM9_ESM.docx]

**Supplementary Information**

**Additional file 1: Fig. S1.**

**Additional file 1: Fig. S2.** (A) The transfection efficiency of sh-PTBP1 was detected by western blot. Data represent mean ± SD (n = 3, each group). **P* < 0.05, ***P* < 0.01 versus siRNA-NC group. (B) The transfection efficiency of sh-circRNA_001160 was detected by qRT-PCR. Data represent mean ± SD (n = 3, each group). **P* < 0.05, ***P* < 0.01 versus siRNA-NC group. (C) The transfection efficiency of miR-195-5p was detected by qRT-PCR. Data represent mean ± SD (n = 3, each group). ***P* < 0.01 versus pre-NC group; ^##^*P* < 0.01 versus anti-NC group. (D) The overexpression transfection efficiency of ETV1 was detected by western blot. Data represent mean ± SD (n = 3, each group). ***P* < 0.01 versus ETV1-NC group. (E) The silent transfection efficiency of ETV1 was detected by western blot. Data represent mean ± SD (n = 3, each group). ***P* < 0.01 versus siRNA-NC group. (F) The expression of miR-195-5p was measured after knockdown of circRNA_001160 by qRT-PCR. Data represent mean ± SD (n  =  5, each group). ***P*  <  0.01 versus sh-NC group. (G) The expression of circRNA_001160 was measured by qRT-PCR. Data represent mean ± SD (n  =  5, each group). **P* <  0.05 versus pre-NC group; ^#^*P*  <  0.05 versus anti-NC group.

**Additional file 1: Fig. S3.** (A) MiRNA microarray analysis of total RNAs isolated from sh-NC and sh-circRNA_001160 cells. Red indicates high relative expression and green indicates low relative expression. (B) FISH showed co-localization between circRNA_001160 and miR-195-5p in GEC cells (red, circRNA_001160; green, miR-195-5p; blue, DAPI nuclear staining). Scale bar represents 20 μm.

**Additional file 1: Fig. S4.** (A) Transcription factor microarray analysis of total RNAs isolated from pre-NC and pre-miR-195-5p cells. Red indicates high relative expression and green indicates low relative expression. (B) Effects of ETV1 on ZO-1, occludin, and claudin-5 expression levels determined by western blot. Data represent mean ± SD (n = 3, each). ***P* < 0.01 versus ETV1-NC group; ^##^*P* < 0.01 versus sh-NC group. (C) Effects of ETV1 on ZO-1, occludin, and claudin-5 expression levels and distribution determined by imunofluorescence staining (n = 3, each). ZO-1, occludin, and claudin-5 (green) were labeled with secondary antibody against anti-ZO-1, anti-occludin, and anti-claudin-5 antibody, respectively, and nuclei (blue) were labeled with DAPI. Scale bar represents 20 μm.
